# Hypoxia-induced long non-coding RNA LINC00460 promotes p53 mediated proliferation and metastasis of pancreatic cancer by regulating the miR-4689/UBE2V1 axis and sequestering USP10

**DOI:** 10.7150/ijms.87833

**Published:** 2023-09-04

**Authors:** Ronghao Zhang, Xinjing Wang, Xiayang Ying, Yishu Huang, Shuyu Zhai, Minmin Shi, Xiaomei Tang, Jia Liu, Yusheng Shi, Fanlu Li, Weishen Wang, Xiaxing Deng

**Affiliations:** 1 Department of General Surgery, Pancreatic Disease Center, Ruijin Hospital, Shanghai Jiao Tong University School of Medicine, Shanghai, China.; 2 Research Institute of Pancreatic Diseases, Shanghai Jiao Tong University School of Medicine, Shanghai, China.; 3 State Key Laboratory of Oncogenes and Related Genes, Shanghai, China.; 4 Institute of Translational Medicine, Shanghai Jiao Tong University, Shanghai, China.

## Abstract

Long non-coding RNAs are considered to be key regulatory factors of oncogenesis and tumor progression. It is reported that LINC00460 plays the role of oncogene in some tumors. However, LINC00460's role and mechanism of action in pancreatic cancer have not yet been fully elucidated. We identified LINC00460 by analyzing data from the Gene Expression Omnibus database. The role of LINC00460 in proliferation and metastasis was examined using CCK8, colony formation, wound healing, and transwell assays. The potential mechanisms of LINC00460 in regulating mRNA levels were elucidated by RNA pull-down, RNA immunoprecipitation, Chromatin immunoprecipitation, Co-immunoprecipitation, and Immunofluorescence assays. The results showed that LINC00460 was upregulated in pancreatic cancer cells and tissues. Highly expressed LINC00460 is significantly related to short survival of pancreatic cancer patients. Inhibition of LINC00460 attenuated pancreatic cancer cell proliferation and metastasis, whereas its overexpression reversed this effect. Mechanically, LINC00460 is induced by hypoxia, through binding of the hypoxia-inducible factor 1-α in the promoter region of LINC00460. Furthermore, LINC00460 functioned as an miR-4689 sponge to regulate the downstream target gene UBE2V1, enhancing the stability of mutant p53 in pancreatic cancer cells. LINC00460 also further promotes pancreatic cancer development by sequestering USP10, a cytoplasmic ubiquitin-specific protease that deubiquitinates p53 and enhances its stability. Collectively, our study demonstrated that LINC00460 is a hypoxia-induced lncRNA that plays the role of oncogene in pancreatic cancer by modulating the miR-4689/UBE2V1 axis, sequestering USP10, and ultimately enhancing the stability of mutant p53.

## Introduction

Pancreatic ductal adenocarcinoma (PDAC) is a highly aggressive tumor, and in the United States, it ranks third among cancer-related mortality 1. This disease is usually asymptomatic in the early stages and is characterized by late stage and metastasis when diagnosed. Although treatment methods have greatly improved in recent years, chemotherapy remains the only opportunity for most patients with pancreatic cancer 2. Unfortunately, many of them are insensitive to chemotherapy and quickly develop resistance to chemotherapy drugs 3. Therefore, finding new therapeutic targets for pancreatic cancer has become an urgent problem.

Long non-coding RNAs (lncRNAs) are RNA transcripts that contain more than 200 nucleotides but do not encode proteins 4. lncRNAs play a crucial role in epigenetic regulation and differentiation. Furthermore, a growing number of studies have shown that lncRNAs are involved in the pathological process of cancer 5, 6. LINC00460 has been reported to participate in proliferation, metastasis, and invasion of various tumor types. For example, LINC00460 plays the role of oncogene by sponging miR-342-3p and further regulates AGR2 expression to promotes the progression of hepatocellular carcinoma 7. In gastric cancer, LINC00460 co-operated with EZH2 and LSD1 to inhibit expression of CCNG2 and further enhances proliferation 8. Further evidence indicates that LINC00460 is the main regulator of molecular pathways in breast cancer and can influence clinical outcome 9. More interestingly, LINC00460 binds directly to PRDX1, promoting PRDX1 into the nucleus and promoting epithelial-mesenchymal transformation (EMT) in squamous cell carcinoma of the head and neck 10. Nevertheless, the specific role of LINC00460 in pancreatic cancer remains unclear.

Previous studies have revealed that hypoxia is frequently associated with tumor progression 11-13. Due to the dense extracellular matrix of pancreatic tumors, pancreatic cancer cells are chronically hypoxic 14, 15. Under hypoxic conditions, hypoxia inducible factor 1 alpha (HIF-1 alpha) is activated and then promotes the transcription of its downstream target gene 16, 17. Recently, the impact of hypoxia on the expression of lncRNAs has been a focus of attention. Some hypoxia-responsive lncRNAs play significant roles in tumor development; for instance, hypoxia-induced overexpression of lncRNA-BX111 enhanced the metastatic ability of pancreatic cancer. Furthermore, hypoxia promoted EMT, which could be partially inhibited by the downregulation of BX111^18^. lncHILAR is a hypoxia-induced LncRNA that plays an important regulatory role in renal cell carcinoma and promotes the invasion of tumor cells 19. MIR210HG is a key hypoxia-regulated lncRNA involved in glioma invasion, cancer stemness and temozolomide resistance 20. In summary, pancreatic cancer occurs in a low oxygen environment, and the abnormal expression of lncRNAs induced by hypoxia in pancreatic cancer requires further research.

It is reported that miR-4689 decreased tumor progression in colorectal cancer 21, nevertheless, its role in pancreatic cancer is still unclear. Ubiquitin conjugating enzyme 2 variant1 (UBE2V1), mainly functions with its co-factor UBC13, plays a significant role in the formation of protein polymers through k63-dependent ubiquitination 22. UBE2V1 is upregulated in colorectal cancer and melanoma and is associated with poor prognosis 23, 24. However, its role in pancreatic cancer remains unclear. Ubiquitin specific peptidase 10 (USP10) is a highly conserved deubiquitinating enzyme with complex roles in tumorigenesis. For example, overexpression of USP10 stabilizes Krüppel-like factor 4 and suppresses lung tumorigenesis 25, whereas USP10 deubiquitinates NLRP7 and promotes colorectal cancer progression by activating the immunosuppressive state 26. The roles and specific mechanisms of USP10 in pancreatic cancer require more detailed research 27.

Wild-type p53 is a tumor suppressor whose expression is closely linked to the DNA damage repair and cell cycle regulation. P53 expression is regulated by post-translational modifications, including but not limited to, methylation, acetylation, phosphorylation and ubiquitination 28. P53 is mutated in multiple tumors, and the mechanism of gain-of-function mechanism is an important factor in the induction of tumor progression by mutant p53^29^-^31^. P53 mutation is one of the four most common mutations in pancreatic cancer; thus, most pancreatic cancer cells contain mutant p53, whose upregulation subsequently promotes tumorigenesis 32, 33. The mechanism of upregulation of mutant p53 protein in pancreatic cancer requires further investigation.

In this study, we discovered that LINC00460 is upregulated in pancreatic cancer, based on bioinformatic analysis and specimen information in our center. The high expression of LINC00460 promotes proliferation and metastatic capacity. Furthermore, upregulation of LINC00460 in pancreatic cancer is induced by HIF-1α under hypoxic conditions. Mechanistically, LINC00460 plays the role of oncogene by acting as a sponge of miR-4689, activating its downstream target, UBE2V1, and sequestering USP10 to promote the proliferation and metastasis of pancreatic cancer. Remarkably, both UBE2V1 and USP10 participate in the regulation of p53 by enhancing the stability of p53, ultimately promoting the proliferation and metastatic capacity of pancreatic cancers with mutant p53. Altogether, our study demonstrates that LINC00460 may be a potential biomarker for pancreatic cancer.

## Materials and methods

### Patients and specimens

Sixty pairs of pancreatic cancer tissues and their adjacent normal tissues were collected from our center. The patients who participated met the following criterion: 1 the pathological diagnosis was pancreatic cancer; 2 no preoperative chemotherapy; 3 with complete medical history data. All participants were informed and agreed to participate in the study, and the study has been reviewed by the ethics committee.

### Bioinformatic analysis

Obtaining LncRNA expression matrix from GEO database (GSE16515, GSE46234, GSE17890 and GSE15471). GEO2R online tool was applied to perform the differential analysis. The GEPIA website based on TCGA database is used to analyze the expression and prognosis data of LINC00460. The online prediction tool lncLocator was used to locate and predict LINC00460. Prediction of binding sites of HIF-1a and LINC00460 using JASPAR online tool.

### Cell culture

All cell lines (Bxpc3, CFPAC, Patu8988, PANC1, MiaPaCa2, Capan2 and HPNE) were purchased from the Chinese Academy of Sciences. Cells were cultured in RPMI 1640 or DMEM medium supplemented with 10% fetal bovine serum and 1% penicillin streptomycin solution. The incubator with 5% co2 at 37 ° C is used to culture cells. Cells were cultured under hypoxia in the environment of 1% O2 and 5% CO2 at 37 ℃.

### qRT-PCR analysis

Total RNA and complementary DNA (cDNA) from pancreatic tissues and cell lines were isolated and obtained using AG RNAex Pro Reagent (Agbio, China). RT-PCR was performed using Evo M-MLV reverse transcription kit (Agbio, China). GAPDH was used as a routine control and U6 as a control for miRNA. All primer sequences are listed in Table S1.

### Cell transfection

Lipofectamine™ 3000 (Invitrogen, USA) or Hilymax reagent (Dojindo, Japan) were used to transfect siRNAs, miRNAs mimics, inhibitors and target plasmids into pancreatic cancer cell lines. Lentiviral plasmids required for stable transfection are synthesized by inserting the sequence of interest into the expression vector pcDNA3.1 (Bioegene, Shanghai, China). HEK-293T cells in good growing condition were seeded in a 6-well plate at a density of 2 × 105 cells per well and cultured to 70-80% confluence. 1ug lentiviral plasmids, 0.5ug psPAX2, 0.5ug pMD2G and 5ul Lipofectamine™ 3000 or 14ul Hilymax reagent were mixed and diluted in 200ul Opti-medium and waited for 20 min. Then added the mixed solution to the cell culture medium and cultured for 48h. Collected the supernatant virus solution using a 0.22um filter and stored in the -80℃ refrigerator. Pancreatic cancer cells were seeded in 6-well plates (2 × 105 cells per well) and cultured to 70-80% confluence. 2ml of the viral liquid described above was added and cultured for 48h to obtain stable transfectants. Puromycin was used to screen stable transfected cell lines. The siRNAs and miRNAs sequences are listed in Table S2.

### Fluorescenece in situ hybridization (FISH)

CY3-labeled LINC00460 probes were designed and synthesized by RiboBio (Guangzhou, China). FISH assay was performed with a Ribo Fluorescent In Situ Hybridization Kit. In short, cells were fixed with 4% paraformaldehyde for 10min followed by washing with PBS for 3 times. Fixed cells were further permeabilized with 0.5% Triton X-100 and then washed with PBS for 3 times. Cells were incubated with 40 nM FISH probe (Ribobio) in hybridization buffer (100 mg/ml dextran sulfate and 10% formamide in 2× SSC) at 37 °C overnight. Then, cells were washed with 4× SSC for 3 times and 2× SSC (0.3 M NaCl and 0.03 M Na3 citrate (pH 7.0)) once and 1× SSC once. The nucleus was stained with DAPI (Thermo Fisher Scientific, USA). Observation of subcellular location of LINC00460 using confocal fluorescence microscope (Zeiss, Germany).

### Western blot assay

RIPA reagent was used to lyse tissues and cells to extract total protein. The protein is transferred to PVDF membrane through gel electrophoresis and membrane transfer. Then block the membranes with quick block fluid for 15min and incubated with primary antibodies at 4 °C for 12h. After that, incubated with secondary antibodies. Western blot imaging analyzer (Thermo Fisher Scientific, USA) was used to analyse the protein expression. Antibodies used are listed in Table S3.

### Cell counting kit-8 (CCK8) assay

Transfected CFPAC, Panc1, MiaPaCa2 and Capan2 cells were laid in a 96-well plate at a density of 1.0 × 103 cells per well and cultured for 24h. Then 100µL fresh medium and 10µL CCK-8 solution (Dojindo, China) were added. Lastly, cells were placed in the incubator and the absorbance was measured every 3 h. The cell viability was calculated.

### Colony formation assay

PANC1, CFPAC and MiaPaCa2 cells were seeded in a 6-well plate at a density of 1 × 103 cells per well and cultured for 2 weeks. Capan2 cells were seeded in a 6-well plate at a density of 3 × 103 cells per well and cultured for 2 weeks. Cells were fixed with crystal violet for 15 min. Image J software was used to count cell clones.

### Flow cytometry analysis

Transfected cells were placed in a 6-well plate (4 x 104 cells/well) (Corning Corporation, USA) and incubated for 24 h. Then washed cells with PBS twice and then cultured in the medium for 48 h. Digested the cells with EDTA-Trypsin and collected cells, then centrifuged at 1000 rpm for 5 min, suspended with PBS and centrifuged at the same condition twice. Then resuspended with 100ul binding buffer and stained with Annexin-V/7-AAD apoptosis kit for 15min (Vazyme, China).

### Wound healing assay

Transfected cells were laid in a 6-well plate (5x104 cells/well) (Corning Corporation, USA), cultured to 100% confluence and scratched with sterile plastic micropipette tip. Washing with PBS twice, cells were cultured in medium for 24 h. Record the scratch size under an inverted Microscope (Zeiss, Germany). Migration distance was assessed using the Image J software.

### Transwell assay

Transwell chambers (Costar Corporation, USA) were applied to conduct this experiment. 3 × 103 cells were suspended in 200 ul medium, these cells were seeded into the upper chamber. The lower insert was filled with 700μl medium containing 10% FBS. After 48 h of incubation, cells were fixed with crystal violet for 15 min and the cells were imaged and calculated.

### Dual-luciferase reporter assays

A sequence containing the potential binding site of LINC00460 and the 3′UTR of UBE2V1 and the corresponding mutant sequence were synthesized and cloned into the pGL3 luciferase reporter vector (Promega, USA). The LINC00460 promoter fragment containing HRE1 but not HRE2 was synthesized and cloned into the pGL3 luciferase reporter vector (termed HRE1). The LINC00460 promoter fragment containing HRE2 but not HRE1 was synthesized and cloned into the pGL3 luciferase reporter vector (termed HRE2). HEK-293T cells were seeded in 6-well plates (2×105 cells per well) and cultured to 70-80% confluence. 800ng of the mimics or reporter plasmids described above were transfected into the HEK-293T cells. After 48h of transfection, the cells were further processed. Finally, the absorbance was detected using a Dual-Luciferase Reporter Assay System (Promega, USA). The sequences used are listed in table S4.

### RNA pull-down assay

Biotinylated LINC00460 and its corresponding chain were designed and synthesized by Ribobio (Guangzhou, China). These RNAs were incubated with the lysate of CFPAC cells at 4 ℃, and then incubated with agarose beads to pull down the binding proteins At last, mass spectrometry and western blot analysis were applied to protein separation and analysis.

### Tumor xenograft model

Stable transfected MiaPaCa2, PANC1 and CFPAC (1 × 10^7^ cells) were resuspended with 200 ul PBS and injected into the subcutaneous axilla of 6 weeks BALB/c nude mice, respectively. The mice were killed after 4 weeks of injection, and the length, width and weight of tumors were measured. CFPAC (1 × 10^6^ cells) were resuspended in 25 ul PBS and injected into the spleen of 6 weeks BALB/c nude mice. The mice were killed after 4 weeks of injection, and their livers were removed to see if there were metastases. The animal experiments have been reviewed by the ethics committee.

### Immunohistochemical (IHC) analysis

Melt paraffin slices at 65°C for 1 h, and then dewaxed and hydrated with xylene and ethanol of different concentrations. The antigen was retrieved using sodium citrate antigen retrieval solution, then permeabilized with 0.25% TritonX-100 (MCE, USA) and pretreated with DAB stain buffer. Furthermore, slices were incubated with primary antibodies at 4 °C overnight and then incubated with HRP enzyme-labeled goat anti-mouse or rabbit IgG polymer at room temperature. Add DAB color solution and dye with hematoxylin. Lastly, dehydration and transparency with different concentrations of xylene and ethanol. Representative pictures were taken by scanner.

### Chromatin immunoprecipitation (ChIP) assay

Pierce Magnetic ChIP Kit (Thermo Fisher Scientific, USA) was applied to perform the ChIP analysis. Crosslink Panc1, CFPAC and MiaPaCa2 cells with 4% paraformaldehyde and terminate with glycine. Using ultrasound to disintegrate the nuclear lysate and incubate it with HIF-1α antibody. Rabbit IgG antibody was used as control. Primers used in CHIP assay are listed in Table S1.

### RNA immunoprecipitation (RIP) assay

Magna RIP RNA-Binding Protein Immunoprecipitation Kit (Millipore, Billerica, MA, USA) was applied to conduct the RIP assay. Briefly, Panc1, CFPAC and MiaPaCa2 cells (1 × 107 cells) were digested and suspended in 200ul cell lysis buffer and stored in the -80℃ refrigerator. Then washed the immunoprecipitated magnetic beads twice with RIP wash buffer and incubated with antibodies for 30 min at room temperature and then washed with RIP wash buffer three times. Incubated the beads and cell lysis buffer in 4℃ overnight and washed for 6 times. At last, purified the RNA on the beads and collected the sediment. The coprecipitated RNA fragments were used for qRT-PCR detection.

### Co-immunoprecipitation (CoIP) assay

Protein G, immunoprecipitation kit (Roche, Switzerland) was used to perform CoIP analysis. Panc1 and CFPAC cells were washed with PBS twice and then lysed with NP40 buffer for 10 min. Centrifuged at 12000r for 10 min and collected the supernatant. Supernatant incubated with protein G agarose and purpose antibodies at 4°C overnight. Then protein G agarose was washed with wash buffer 1, 2 and 3. Lastly, the agarose was washed with loading buffer and subjected to western blot assays.

### Immunofluorescence (IF) assay

PANC1, CFPAC MiaPaCa2 and Capan2 cells were planted in the 8-well IF plate and cultured overnight. Cells were washed by PBS twice and fixed by paraformaldehyde 15min on ice and washed with PBS twice, and then ruptured the cell membranes by 0.25% TritonX-100 (Sigma, USA). After that, 3% BSA blocking for 30 min. P53 and USP10 (Proteintech, USA) antibodies (1:100) incubated at 4°C for 12 h and washed with TBST, then incubated with secondary antibody (Servicebio, China) for 1h at room temperature and washed with TBST for 3 times. At last, staining with DAPI (Servicebio, China) for 15min and representative pictures were taken by confocal fluorescence microscope.

### Statistical analysis

Data analysis is completed by the SPSS (version 22.0) and GraphPad Prism 8.0. The statistical analysis of the difference between the two groups was completed by paired t-test. The survival analysis of patients is represented by Kaplan Meier curve. COX regression model was used to analyze the expression of related genes and their impact on the survival of patients. P < 0.05 is considered statistically different.

## Results

### LINC00460 is highly expressed in pancreatic cancer and is related to poor prognosis

**Figure 1:**
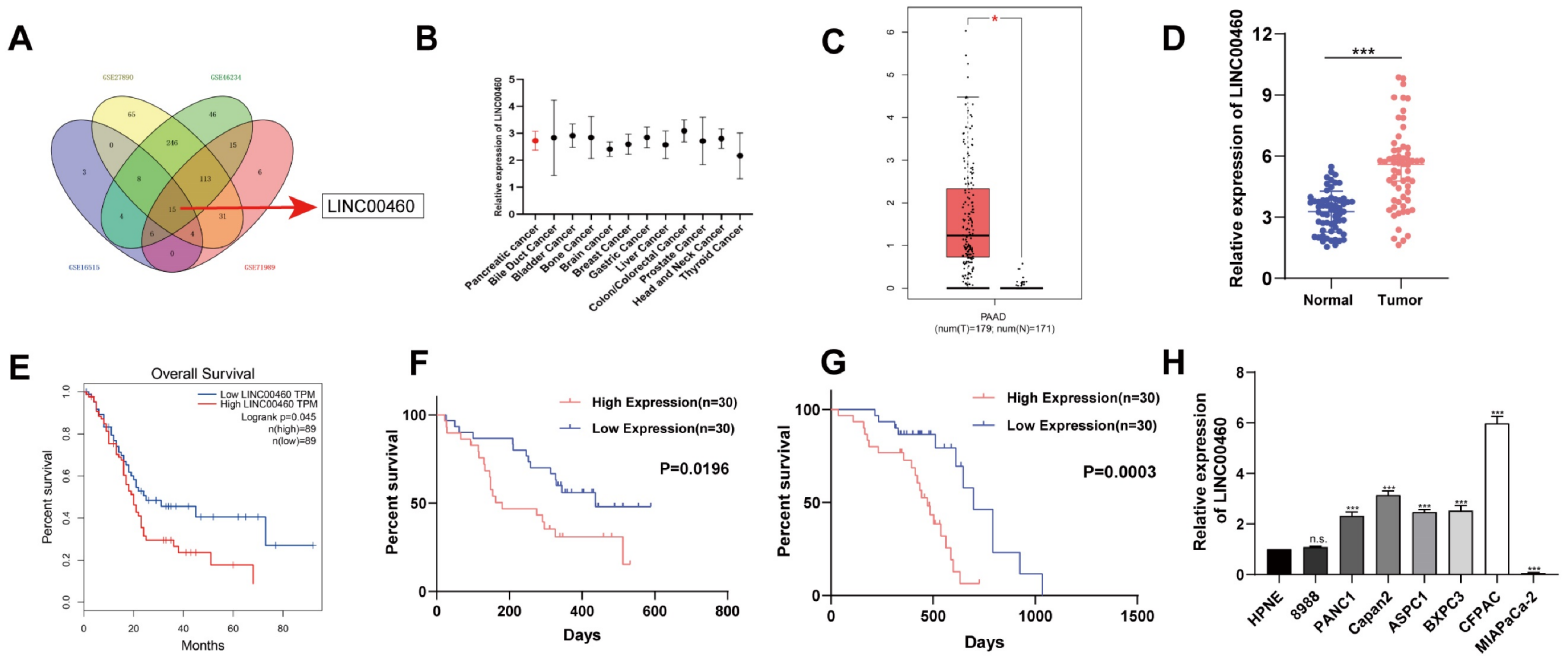
** LINC00460 is highly expressed in pancreatic cancer and is associated with poor prognosis**. **(A)** Venn diagram shows that LINC00460 is highly expressed in GSE16515, GSE27890, GSE46234 and GSE71989 database. **(B)** LINC00460 is highly expressed in other cancers. **(C)** Differential expression of LINC00460 in tumor tissues and normal tissues in pancreatic cancer from TCGA. **(D)** LINC00460 expression in tumor tissues and normal tissues in our center. **(E)** Overall survival analysis from TCGA database. **(F)** Disease free and **(G)** overall survival analysis in our center. **(H)** Differential expression of LINC00460 in various pancreatic cancer cell lines. *P<0.05; ***P<0.001; n.s. no significance.

To find potential lncRNAs that might be involved in the progression of pancreatic cancer, differential expression analysis was performed using data from GEO datasets (GSE16515, GSE27890, GSE46234, and GSE71989). We found 15 lncRNAs, including LINC00460, were expressed differently in normal and pancreatic cancer tissues (Figure 1 A). Furthermore, we identified that LINC00460 was upregulated in other tumors and may be a latent oncogene in pancreatic cancer (Figure 1 B). Subsequently, GEPIA, an analytical tool based on TCGA, was used to verify the expression of LINC00460 in pancreatic cancer and its impact on patient prognosis. We determined that LINC00460 has higher expression in pancreatic cancer tissues than in normal tissues, in both TCGA database and using data from our institute (Figure 1 C, D). Data from TCGA database also suggested that higher expression of LINC00460 was closely related to lower overall survival rate of patients (Figure 1 E). In agreement with this, 30 paired tissue samples from our center also demonstrated that the high expression of LINC00460 was associated with a shorter disease-free survival and overall survival (Figure 1 F, G). Finally, compared with normal pancreatic epithelial cells, LINC00460 expression increased in seven of the eight pancreatic cancer cell lines tested (Figure 1 H). The above evidence suggests that LINC00460 may be a potential oncogene in pancreatic cancer.

### LINC00460 promotes proliferation and metastatic capacity of pancreatic cancer cell lines

**Figure 2:**
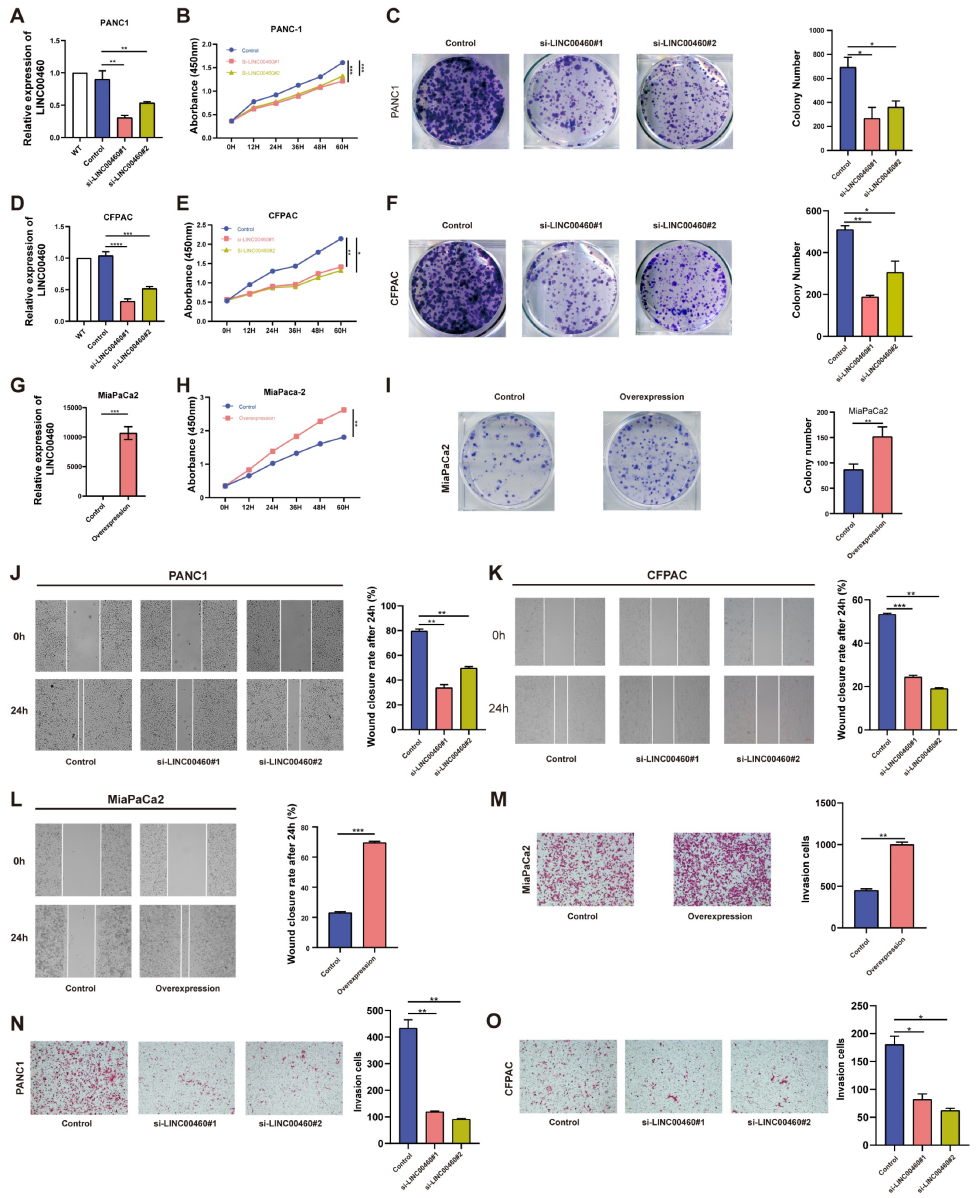
** LINC00460 promotes proliferation and metastasis of pancreatic cancer cells.** Knockdown efficiency of LINC00460 in PANC1 **(A)**, CFPAC **(D)** and overexpression efficiency in MiaPaCa2 **(G)**. Representative results of CCK8 assays in PANC1 **(B)**, CFPAC **(E)**, and MiaPaCa2** (H)**. Representative images and results of colony formation assays in PANC1 **(C)**, CFPAC **(F)** and MiaPaCa2 **(I)** cells. Images and results of wound closure rate after 24h in PANC1** (J)** and CFPAC **(K)** after downregulation of LINC00460 and overexpression of LINC00460 in MiaPaCa2 **(L)**. Representative images and results of transwell assays in PANC1 **(N)**, CFPAC **(O)** and MiaPaCa2 **(M)** cells. *P<0.05; **P<0.01; ***P<0.001; n.s. no significance.

To verify the specific function of LINC00460 in pancreatic cancer, short interfering RNAs were used to reduce the high expression of LINC00460 in the human pancreatic cancer cell lines PANC1 and CFPAC. MiaPaCa2, with low LINC00460 expression, was selected as a cell model for stable overexpression. Knockdown and overexpression efficiencies were verified by qRT-PCR (Figure 2 A, D, G). CCK-8 and colony formation assays were used to examine proliferation ability. Knockdown of LINC00460 in PANC1 and CFPAC cell lines inhibited their proliferation ability (Figure 2 B, C, E, F), while overexpression of LINC00460 in MiaPaCa2 enhanced proliferation (Figure 2 H, I). Subsequently, wound healing (Figure 2 J, K) and transwell assays (Figure 2 N, O) showed that knockdown of LINC00460 reduced the metastasis ability of PANC1 and CFPAC cells. In addition, LINC00460 overexpression in MiaPaCa2 cells increased metastatic potential, indicating that LINC00460 may facilitate metastasis (Figure 2 L, M). Western blot assays were used to verify proliferation and metastasis when LINC00460 was knocked down or overexpressed Figure S2 D). Flow cytometry revealed that knocking down LINC00460 significantly promoted apoptosis of PANC1 and CFPAC cells (Figure S1 A, B), whereas overexpression of LINC00460 inhibited apoptosis (Figure S1 C). Western blot assays using apoptosis-related proteins confirmed this result (Figure S1 D). In addition, as shown by flow cytometry and western blot assays, downregulation of the LINC00460 induced cell cycle arrest (Figure S2 A-C).

**Figure 3:**
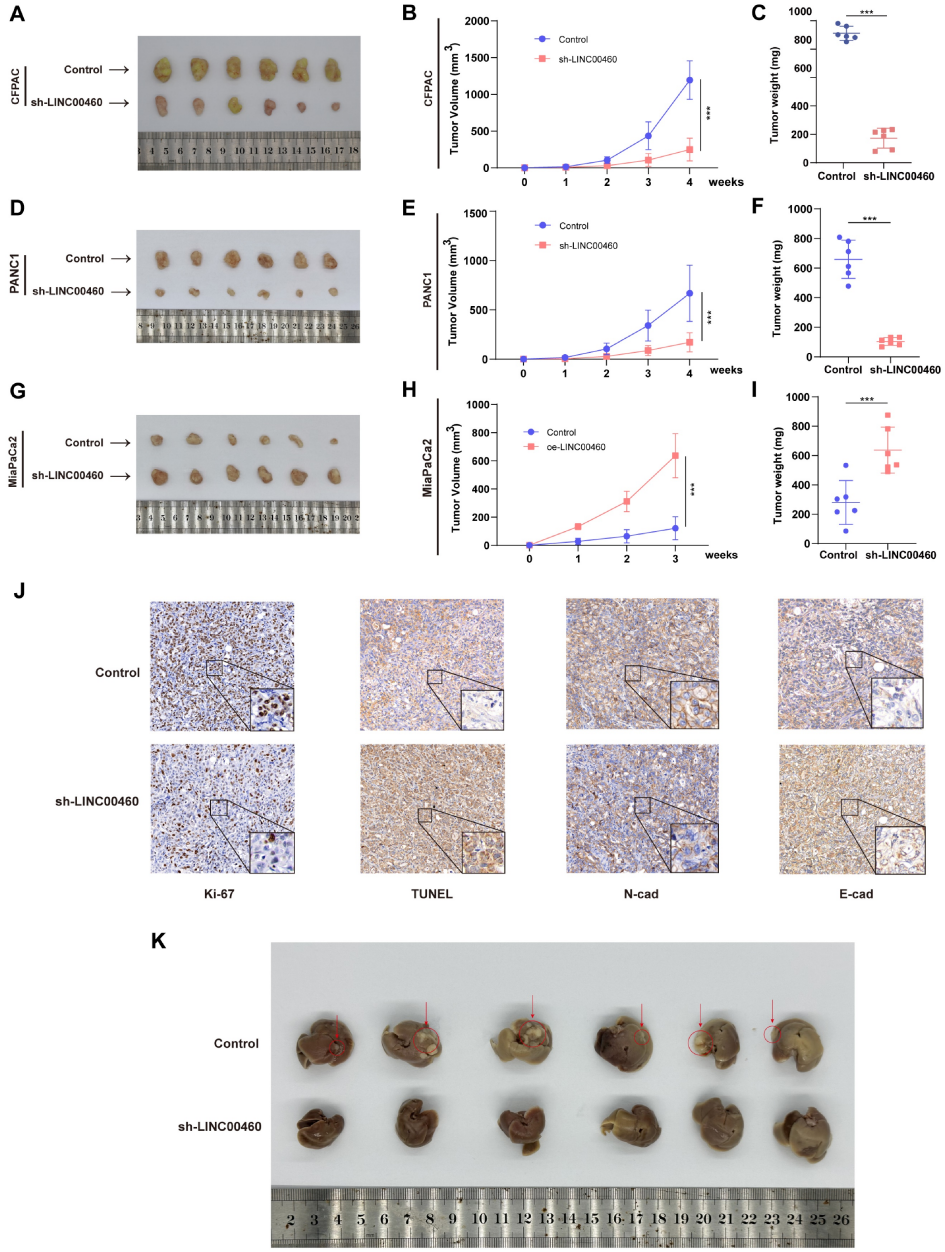
** Knocking down LINC00460 clearly inhibited proliferation and metastasis *in vivo*.** Subcutaneous xenograft tumor generated in nude mice with CFPAC **(A)** and PANC1** (D)** cells knocking down LINC00460 or empty vector. **(G)** Subcutaneous xenograft tumor generated in nude mice with MiaPaCa2 cells overexpressing LINC00460 or empty vector. Xenograft tumor volume of CFPAC **(B)**, PANC1 **(E)** and MiaPaCa2 **(H)**. Xenograft tumor weight of CFPAC **(C)**, PANC1 **(F)** and MiaPaCa2 **(I)**. **(J)** IHC analysis using xenograft tumor tissues derived from CFPAC staining with Ki-67, TUNEL, N-Cad and E-Cad antibodies. **(K)**. 28 days after CFPAC tumor cells were injected into the spleen of nude mice, the mice metastasized tumors in the liver. *P<0.05; **P<0.01; ***P<0.001; n.s. no significance.

The subcutaneous xenograft model was further utilized to verify the biological function of LINC00460 *in vivo*. The results were identical to the *in vitro* experiments, and the tumor volume and weight of the LINC00460 down-regulated group were reduced compared with the control group (Figure 3 A-F), whereas overexpression of LINC00460 promoted tumor growth rate (Figure 3 G-I). IHC analysis of tumor tissues from nude mice revealed that decreased LINC00460 expression resulted in decreased Ki-67 and N-cadherin expression and upregulated TUNEL and E-cadherin expression (Figure 3J). To investigate the effect of LINC00460 on the metastatic function of pancreatic cancer cells *in vivo*, we injected CFPAC cells into the spleen of nude mice. We found that knocking down LINC00460 significantly inhibited the ability of CFPAC cells to metastasize in nude mice (Figure 3K). Overall, our findings indicate that LINC00460 promoted the proliferation and metastatic ability of pancreatic cancer cells, both *in vivo* and *in vitro*.

### Upregulation of LINC00460 in pancreatic cancer is induced by hypoxia

**Figure 4:**
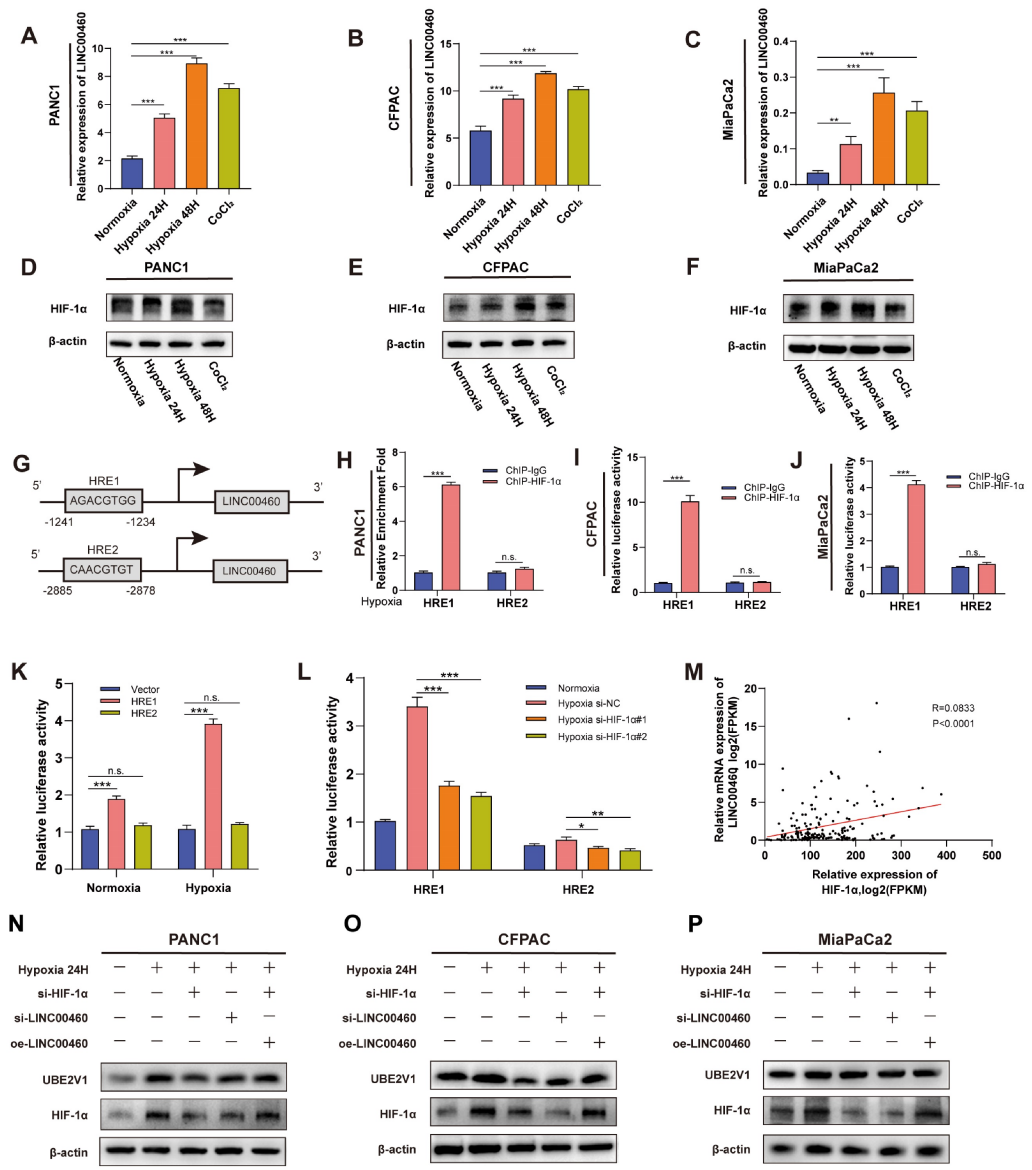
** Upregulation of LINC00460 was induced by hypoxia.** Differential expression of LINC00460 in normoxia, hypoxia 24h, hypoxia 48h and treated with CoCl2 in PANC1 **(A)**, CFPAC **(B)** and MiaPaCa2 **(C)**. Differential expression level of HIF-1α in normoxia, hypoxia 24h, hypoxia 48h and treated with CoCl2 in PANC1 **(D)**, CFPAC **(E)** and MiaPaCa2 **(F)**. **G** Schematic diagram of the binding domain of LINC00460 promoter and hypoxia responsive elements (HREs). ChIP assays with HIF-1α antibody were performed to verify the binding domain of LINC00460 promoter and HRE1 and HER2 under hypoxia in PANC1**(H)**, CFPAC **(I)** and MiaPaCa2 **(J)** cells. **K** CFPAC cells were transfected with LINC00460 promoter-containing reporter vector 48h under normoxia and hypoxia, then a dual-luciferase reporter assay was performed to measure the luciferase activity. **L** Luciferase activity of CFPAC cells transfected with LINC0046 promoter-containing reporter vector and HIF-1α si-RNAs was measured. **M** The correlation analysis between LINC0046 and HIF-1α using data from TCGA. The expression level of HIF-1α and UBE2V1 in PANC1 **(N)**, CFPAC **(O)** and MiaPaCa2 **(P)** cells. *P<0.05; **P<0.01; ***P<0.001; n.s. no significance.

Next, we explored the potential mechanisms of LINC00460 upregulation in pancreatic cancer. Previous studies revealed that hypoxia is closely associated with tumor progression and hypoxia could affect the expression of lncRNAs. To investigate whether upregulation of LINC00460 was induced by hypoxia, we exposed pancreatic cancer cell lines to hypoxia and normoxia conditions for 24 and 48 h or treated with CoCl_2_ for 48 h. qRT-PCR demonstrated that LINC00460 was upregulated in hypoxic environment or after CoCl_2_ treatment (Figure 4 A-C). HIF-1α, the key effector molecule for hypoxia, was also upregulated under these conditions (Figure 4 D-F). siRNAs targeting HIF-1α reduced HIF-1α protein levels (Figure S3 D) and mRNA (Figure S3 E). Data from TCGA database indicate that LINC00460 is positively correlated with HIF-1α, which is consistent with our results (Figure 4 M). To clarify the possible mechanism by which hypoxia induces upregulation of LINC00460, the JASPAR database was used to identify potential hypoxia response elements (HREs) in the LINC00460 promoter region (Figure S3 A). Two high-score HREs were selected by us for further analysis (Figure 4 G). Primers were designed and synthesized for the HREs, and ChIP assays were performed. The results identified that HRE1 might be a potential binding domain in the LINC00460 promoter in pancreatic cancer cell lines (Figure 4 H-J). In addition, luciferase reporter vectors including either the HRE1 or HRE2 sequence were synthesized. The luciferase activity in cells transfected with HRE1 was higher than that in cells with HRE2, and this increase was more pronounced under hypoxic conditions. Thus, HRE1 may be the key binding site for HIF-1α to upregulate LINC00460 (Figure 4 K). SiRNAs were utilized to verify the result. Knockdown of HIF-1α reduced luciferase activity of its binding domain (Figure 4 L). Overall, these results indicated that under hypoxic conditions, HIF-1α induced upregulation of linc00460 in pancreatic cancer, and that this required the HRE1 binding site in the promoter of LINC00460.

To further confirm the relationship between LINC00460 and HIF-1α, we performed western blotting and RT-PCR assays. HIF-1α and UBE2V1 protein levels were upregulated under hypoxic conditions. Knockdown of HIF-1α or LINC00460 both reduced the effect of hypoxia. More interestingly, overexpression of LINC00460 partially reversed this inhibitory effect in pancreatic cancer cell lines (Figure 4 N-P). In addition, knockdown of HIF-1α inhibited LINC00460 expression (Figure S3 F). The hypoxic microenvironment of tumors promotes tumor proliferation and metastasis. The proliferative ability of PANC1 and CFPAC cells were greatly enhanced under hypoxic conditions, while knockdown of LINC00460 and HIF-1α attenuated this effect. Moreover, the decreased proliferative activity resulting from the knockdown of HIF-1α could be partially rescued by overexpressing LINC00460 (Figure S4 A, B). Knockdown of LINC00460 and HIF-1α could also suppress the metastasis of PANC1 and CFPAC cells, whereas overexpression of LINC00460 could partly reverse this inhibitory effect (Figure S4 C-F). In brief, these results suggested that LINC00460 participated in hypoxia-induced proliferation and metastasis of pancreatic cancer.

### LINC00460 functions as a miR-4689 sponge and upregulates UBE2V1 expression in pancreatic cancer

**Figure 5:**
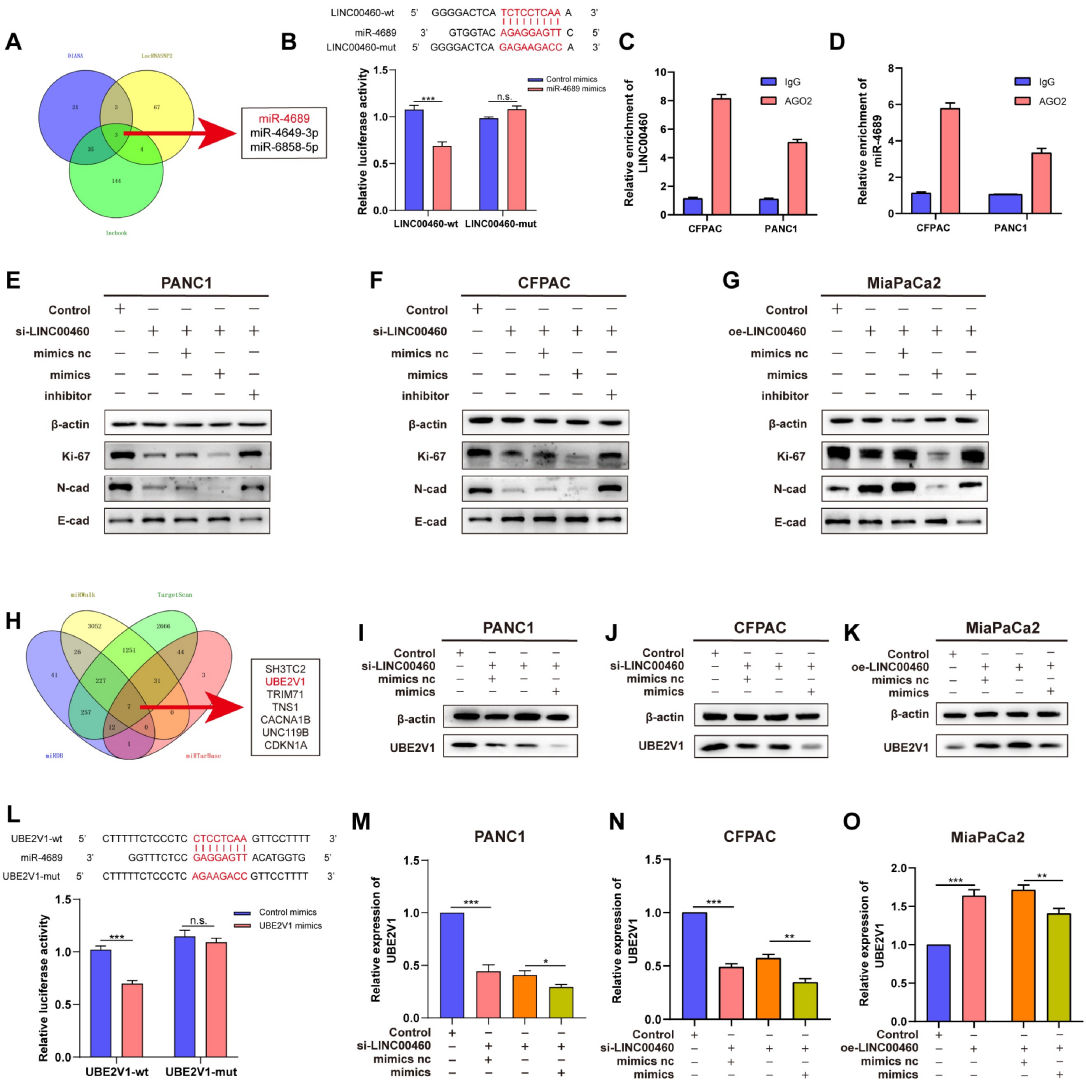
** LINC00460 acts as a miR-4689 sponge and regulated the expression of UBE2V1. A** Three predicted websites DIANA, LncRNASNP2 and lncbook showed that miR-4689, miR-4649-3p, miR-6858-5p might be the potential targets of LINC00460.** B** Dual-luciferase reporter assay was used to verify the combination of LINC0046 and miR-4689. RIP assays using AGO2 and IgG antibodies showed the enrichment of LINC0046** (C)** and miR-4689 **(D)**. The protein levels of Ki-67, N-cadherin and E-cadherin in PANC1 **(E)**, CFPAC **(F)** and MiaPaCa2 **(G)** cell lines. **H** Seven predicted genes of miR-4689 using miRDB, miRWalk, TargwtScan and miRTarBase. UBE2V1 protein level in PANC1 **(I)**, CFPAC **(J)** and MiaPaCa2 **(K)**. **L** Dual-luciferase reporter assay showed that miR-4689 is combined with UBE2V1. UBE2V1 mRNA expression in PANC1 **(M)**, CFPAC **(N)** and MiaPaCa2 **(O)** cell lines. *P<0.05; **P<0.01; ***P<0.001; n.s. no significance.

The mechanism of action of lncRNAs depends on their subcellular localization. To determine the mechanisms by which LINC00460 promotes the cell proliferation and metastasis in pancreatic cancer, to begin with, we predicted the subcellular localization of LINC00460 using lncLocator, an online prediction tool, and found that LINC00460 was mostly localized in the cytoplasm. Furthermore, FISH assays confirmed the above results (Figure S5 A, B). lncRNAs have been reported to act as competing endogenous RNAs (ceRNAs) that bind miRNAs in the cytoplasm. Therefore, we speculated that LINC00460 might also function as a ceRNA in pancreatic cancer. We searched three databases (DIANA, lncRNASNP2, and lncbook) and identified miR-4689, miR-4649-3p, and miR-6858-5p as potential binding partners for LINC00460 (Figure 5 A). While knocking out or overexpressing LINC00460, we quantified the expression of these three miRNAs by qRT-PCR (Figure S5 C-E). MiR-4689 showed the highest upregulation of the miRNAs when LINC00460 was knocked down and was clearly downregulated when LINC00460 was overexpressed. Therefore, we chose miR-4689 as the main downstream miRNA of LINC00460 for further study. We constructed miR-4689 mimics and inhibitors. A dual-luciferase reporter assay confirmed that LINC00460 binds to miR-4689 (Figure 5 B). RIP assay further validated the mutual binding of LINC00460 and miR-4689 in PANC1 and CFPAC cells (Figure 5 C, D). We then performed a complement experiment to verify the interrelationship between miR-4689 and LINC00460, and we found that miR-4689 can partially compensate for the changes in Ki-67, N-cadherin and E-cadherin proteins caused by knocking down LINC00460 in PANC1, CFPAC and MiaPaCa2 cancer cells (Figure 5 E-G).

Using the miRWalk, TargetScan, miRDB, and miRTarBase databases, we identified 7 potential downstream targets of miR-4689 (Figure 5 H). Further qRT-PCR experiments showed that the expression of UBE2V1 changed most significantly after the addition of mimics or inhibitors of miR-4689 (Figure S5 F). Moreover, the expression level of UBE2V1 was positively correlated with the expression of LINC00460, so we chose UBE2V1 as the underlying target of miR-4689 for further experiments (Figure S5 G, H). A dual-luciferase reporter assay revealed that UBE2V1 was a direct target of miR-4689 as well (Figure 5 L). Furthermore, UBE2V1 mRNA (Figure 5 M, N) and protein levels (Figure 5 I, J) decreased in cells transfected with miR-4689 mimics or following knockdown of LINC00460, whereas protein levels increased in MiaPaCa2 cells (Figure 5 K, O). We conclude that LINC00460, as the sponge of miR-4689, regulates its downstream target UBE2V1. Proliferation and metastasis of pancreatic cancer may therefore be mediated by the LINC00460/miR-4689/UBE2V1 axis.

### UBE2V1 regulates the proliferation and metastasis in pancreatic cancer and regulates p53 stability

**Figure 6:**
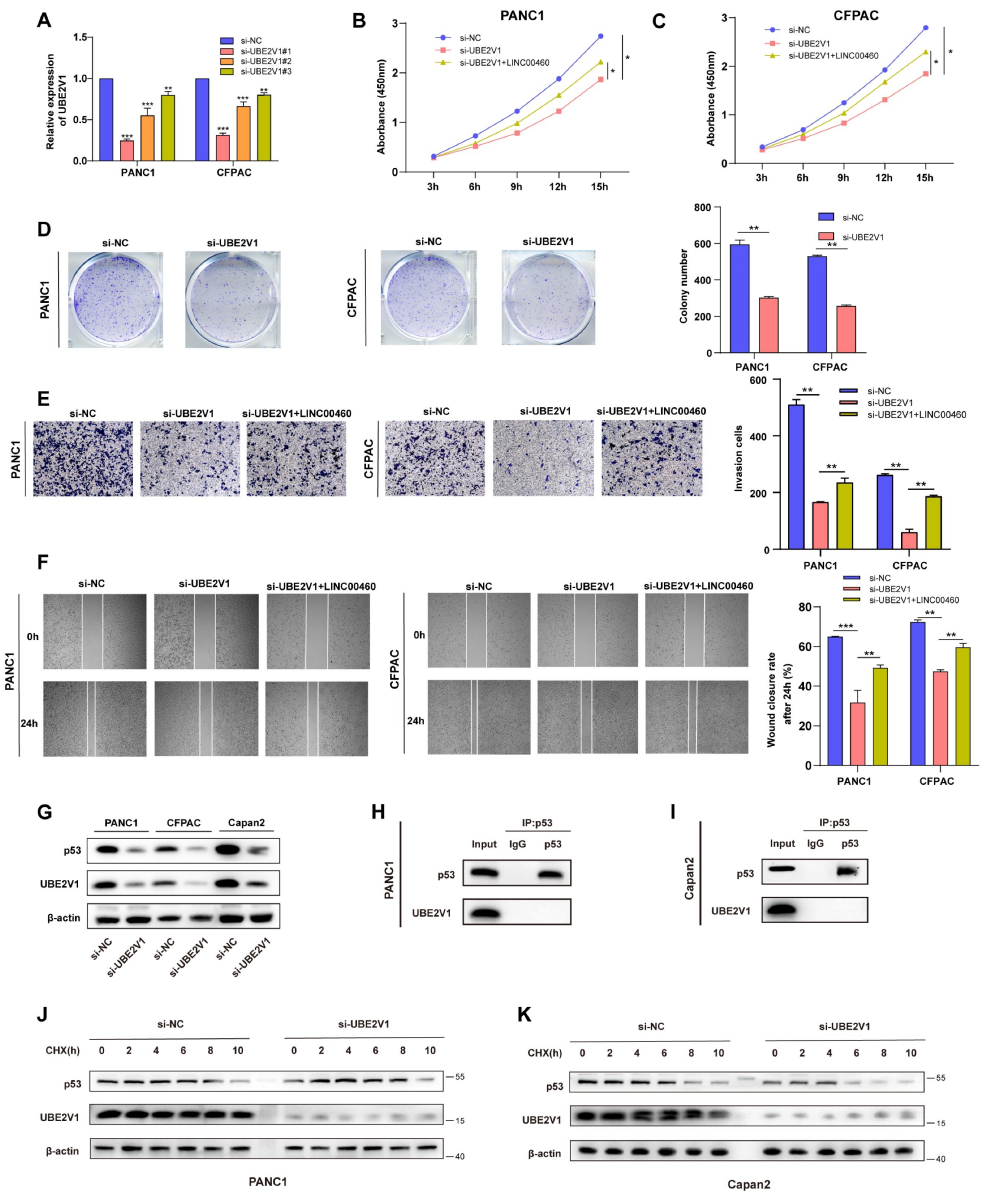
** UBE2V1 regulates proliferation and metastasis in pancreatic cancer and stabilizes p53. (A)** The expression of UBE2V1 transfected UBE2V1 siRNAs. CCK8 assay results in PANC1 **(B)** and CFPAC** (C)** cell lines.** (D)** Representative images and results of colony formation assays in PANC1 and CFPAC. **(E)** Representative images and results of transwell assays in PANC1 and CFPAC. **(F)** Representative images and results of wound healing assays in PANC1 and CFPAC. **(G)** UBE2V1 and p53 protein levels in PANC1, CFPAC and Capan2 cell lines. CoIP assays using p53 antibody to verify the combination of p53 and UBE2V1 in PANC1 **(H)** and Capan2** (I)**. Protein levels of p53 and UBE2V1 treated with CHX for 0, 2, 4, 6, 8 and 10 hours in PANC1 **(J)** and Capan2** (K)**. *P<0.05; **P<0.01; ***P<0.001; n.s. no significance.

UBE2V1, reported as an oncogene, works with its co-factor UBC13, a K63-Ub specific E2 enzyme. However, the function and mechanism of UBE2V1 in pancreatic cancer are unknown. To explore UBE2V1's effect on pancreatic cancer, siRNAs that can reduce the level of UBE2V1 protein and mRNA was used for subsequent experiments (Figure 6 A, G). CCK-8 and colony formation assays indicated that down-regulation of UBE2V1 reduced the proliferation ability of pancreatic cancer cell lines, while overexpression of LINC00460 could partly rescue this effect (Figure 6 B-D). Wound healing and transwell assays also revealed that knockdown of UBE2V1 prevented metastasis, and that upregulation of LINC00460 promoted metastatic ability (Figure 6 E, F). Previous studies have reported that UBE2V1 and, UBC13, could elicit K63-dependent p53 ubiquitination. Unlike conventional ubiquitinated proteins which ubiquitinate and degrade p53, UBE2V1, and UBC13 attenuate Hdm2-induced p53 polyubiquitination and increase its stability (34. Here, we examined whether UBE2V1 could attenuate Hdm2-induced p53 polyubiquitination and enhance the stability of either wild-type or mutant p53 proteins, in pancreatic cancer. The wild-type p53 cell line, Capan2 and two mutant p53 cell lines, PANC1 and CFPAC, were selected Figure S10 A-E). Knockdown of UBE2V1 decreased the expression of mutant and wild-type p53 in pancreatic cancer cells (Figure 6 G). To identify the relationship between UBE2V1 and p53 protein, we conducted Co-IP assays to detect the direct interaction of the two proteins and found that UBE2V1 does not interact directly with either wild-type or mutant p53 (Figure 6 H, I). Additionally, to determine whether UBE2V1 influences p53 stability in pancreatic cancer cells, we treated PANC1 and Capan2 cells with cycloheximide. The results indicated that p53 was degraded faster when UBE2V1 was knocked down in PANC1 and Capan2 cell lines (Figure 6 J, K). In short, these experiments showed that UBE2V1 could stabilize both wild-type and mutant p53. We also explored the biological functions of UBE2V1 in the Capan2 cell line. The knockdown efficiency was tested by qRT-PCR (Figure S6 A).

Furthermore, CCK8 and colony formation assays revealed that downregulation of UBE2V1 promoted the proliferation of Capan2 cells (Figure S6 B, C). Wound healing and transwell migration assays also identified that downregulation of UBE2V1 enhanced the metastatic activity of Capan2 cells (Figure S6 D, E). The above results indicated that the biological function of UBE2V1 in pancreatic cancer cells is p53 context dependent, with UBE2V1 acting as an oncogene in mutant p53 cell lines, while functioning as a tumor suppressor in wild-type p53 cell lines. In summary, UBE2V1, the downstream target of miR-4689, enhances the stability of p53 in pancreatic cancer and further promotes proliferation and metastasis in mutant p53 cell lines.

### LINC00460 regulates proliferation and metastasis by sequestering USP10

**Figure 7:**
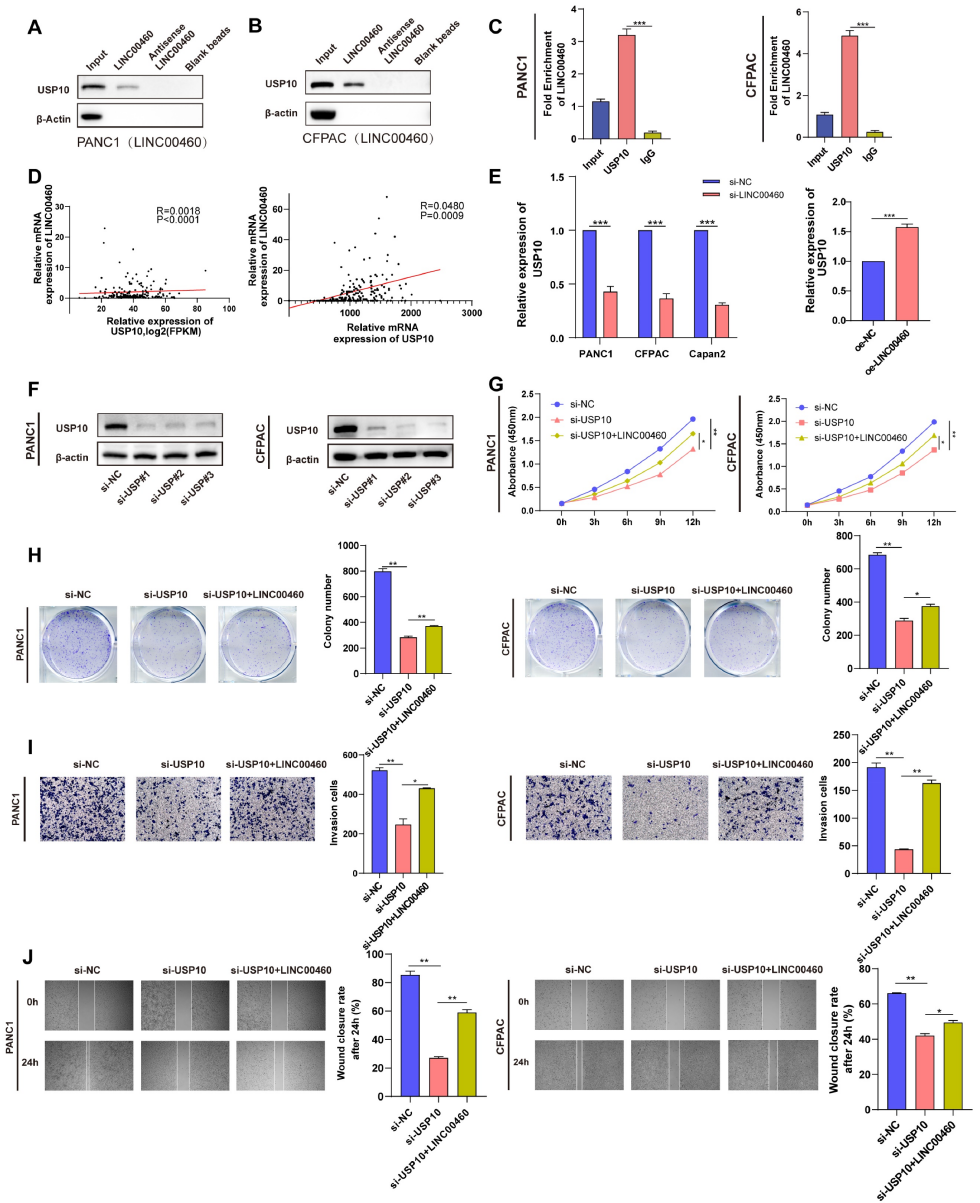
** USP10 directly binds to LINC00460 and regulate proliferation and metastasis of pancreatic cancer cells.** Protein level of USP10 in samples enriched by biotinylated LINC00460, anti-LINC00460 and beads in PANC1 **(A)** and CFPAC **(B)**. **(C)** RIP assays using USP10 antibody to verify the combination between USP10 and LINC00460 in PANC and CFPAC. **(D)** Correlation analysis of LINC00460 and USP10 using data from TCGA and our center.** (E)** USP10 mRNA expression in PANC1, CFPAC and Capan2 cells transfected siRNAs and MiaPaCa2 cell overexpressed LINC00460. **(F)** USP10 protein level in PANC1 and CFPAC transfected siRNAs. **(G)** CCK8 assay results in PANC1 and CFPAC. **(H)** Representative images and results of colony formation assay in PANC1 and CFPAC. **(I)** Representative images and results of transwell assay in PANC1 and CFPAC. **(J)** Representative images and results of wound healing assay in PANC1 and CFPAC. *P<0.05; **P<0.01; ***P<0.00; n.s. no significance.

To additionally investigate the possible mechanism of LINC00460 promoting the progression of pancreatic cancer, we conducted RNA pull-down combined with mass spectrometry. The results showed that the USP10 protein may bind directly to LINC00460. A western blot assay using samples enriched with biotinylated LINC00460 confirmed this (Figure 7 A, B). We then performed RIP assays with a USP10 antibody and verified that there is a direct combination between LINC00460 and USP10 (Figure 7 C). Further correlation analysis showed that LINC00460 expression correlated positively with USP10 expression (Figure 7 D). Additionally, USP10 is highly expressed in pancreatic cancer tissues (Figure S7 A, B) and high expression of USP10 is closely related to poor prognosis in pancreatic cancer, including disease-free survival rates (Figure S7 D, E). The expression of USP10 decreased when LINC00460 was knocked down and vice versa (Figure 7 E). The expression of USP10 was knocked down by siRNAs and verified the knockdown efficiency by qRT-PCR (Figure S8 A, B) and western blot assays (Figure 7 F). Next, we explored the biological function of USP10. CCK8 and colony formation assays suggested that USP10 downregulation decreased cell proliferation, while overexpression of LINC00460 partly rescued this effect (Figure 7 G, H). We also conducted wound healing and transwell migration assays to explore the effect of USP10 on the metastatic capacity of cancer cells (Figure 7 I, J). In brief, we found that USP10 may be a direct-binding partner for LINC00460, and that overexpression of LINC00460 can partially reverse the inhibition of downregulation of USP10 on cell proliferation and metastasis in PANC1 and CFPAC.

### Knockdown of USP10 promotes the translocation of p53 out of the nucleus and accelerates its degradation

**Figure 8:**
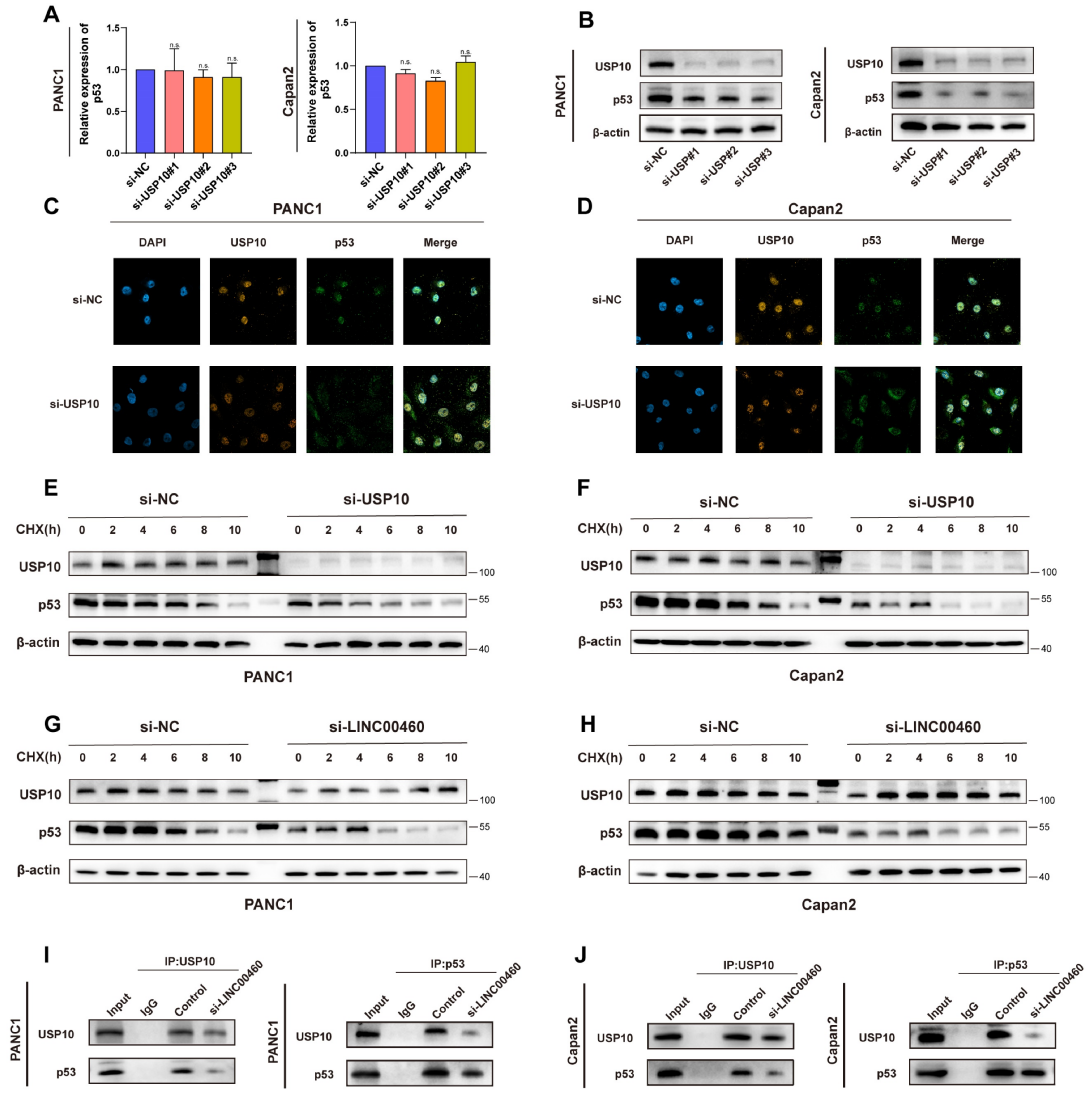
** USP10 regulates the location and stability of p53 protein. (A)** The expression of p53 mRNA while knocking down USP10. **(B)** The protein level of USP10 and p53 while knocking down USP10. **(C)** Location of p53 while knocking down USP10 or not in PANC1. **(D)** Location of p53 while knocking down USP10 or not in Capan2. **(E)** Protein level of p53 while knocking down USP10 or not in PANC1. **(F)** Protein level of p53 while knocking down USP10 or not in Capan2. **(G)** Protein level of p53 while knocking down LINC00460 or not in PANC1. **(H)** Protein level of p53 while knocking down LINC00460 or not in Capan2. **(I)** CoIP assays using USP10 and p53 antibodies to verify the combination of USP10 and p53 while LINC00460 was knocking down or not in PANC1. **(J)** CoIP assays using USP10 and p53 antibodies to verify the combination of USP10 and p53 while LINC00460 was knocking down or not in Capan2. n.s. no significance.

The role of USP10 in pancreatic cancer is unclear, accordingly, we explored the mechanism by which USP10 regulates its downstream targets in pancreatic cancer. Previous studies suggested that USP10 is a deubiquitinating enzyme and modulates the stability of both wild-type and mutant p53(35. Here, we speculated that USP10 functions as a deubiquitinating enzyme in pancreatic cancer as well, inhibiting the p53 ubiquitination, and eventually stabilizing p53. We used PANC1 and Capan2 cell line in the following experiments. Downregulation of USP10 reduced p53 protein levels (Figure 8 B) but not the mRNA levels of TP53 (Figure 8 A), suggesting that changes in p53 protein levels did not occur at the transcriptional level, but at the protein level. Data from TCGA indicated that p53 expression positively correlates with USP10 expression Figure S7 C). Co-IP assays suggested a direct combination between USP10 and p53 (Figure S8 C, D). The expression and localization of p53 are crucial for its biological function; therefore, we performed immunofluorescence assays to determine its localization. Knocking down USP10 could promote translocation of p53 out of the nucleus in PANC1 and Capan2 cells (Figure 8 C, D). Interestingly, p53 was degraded faster in USP10 knockdown cells compared to control cells (Figure 8 E, F). Knockdown of LINC00460 could also accelerate the degradation of p53 protein (Figure 8 G, H). We then conducted Co-IP assays to verify this result and found that knockdown of LINC00460 weakened the interaction between the USP10 and p53 proteins (Figure 8 I, J). The above results indicated that knockdown of USP10 promotes the translocation of the p53 out of the nucleus and accelerates the degradation of p53. Based on the results above, we examined the biological functions of USP10 in Capan2 cells which express wild-type p53. p53 mRNA did not decrease when USP10 was knocked down in Capan2 cells (Figure S9 A, B). Furthermore, the CCK8 assay showed that knockdown of USP10 in Capan2 increased proliferative activity (Figure S9 C) and wound healing and transwell assays suggested that knockdown of USP10 enhanced metastasis ability (Figure S9 D, E). These results showed that the biological function of USP10 is p53 dependent. USP10 promotes proliferation and metastasis in mutant p53 cell lines while inhibiting proliferation and metastasis in wild-type p53 cell lines.

Thus, we revealed that p53 may be a potential target of USP10, and that inhibition of USP10 promotes the translocation of p53 out of the nucleus and accelerates its degradation. Importantly, the expression of LINC00460 can regulate USP10 levels and further manipulate p53 protein levels, thereby regulating the biological behavior of pancreatic cancer.

## Discussion

In recent years, more and more attention has been paid to pancreatic cancer due to its poor prognosis and high morbidity. Because early pancreatic cancer is usually asymptomatic, diagnosis occurs at later stages which, coupled with its malignancy, means that, for most patients, surgery is not an option, making chemotherapy the only viable choice. The efficacy of chemotherapy for pancreatic cancer has shown little progress over the years; therefore, there is an urgent need to explore new targets for pancreatic cancer. Recently, lncRNAs, which are aberrantly expressed in many tumors, have been reported to play a crucial role in the development of various tumors such as liver cancer (36, gastric cancer 37, breast cancer 38, lung cancer 39, and prostate cancer 40. In this study, we discovered that LINC00460 was up-regulated in pancreatic cancer tissues and cell lines. Further correlation analysis revealed that high expression of LINC00460 is closely related to poor prognosis in patients. Furthermore, biological function studies demonstrated that LINC00460 upregulation increased pancreatic cancer cell proliferation and metastasis. Overall, these results indicated that LINC00460 is an oncogene and potential therapeutic target for pancreatic cancer.

Hypoxia is a common phenomenon in solid tumors, especially pancreatic cancer, and is significantly related to tumor development. Recently, some studies have found that lncRNAs are also regulated by hypoxia, specifically by binding to HIF-1α by HREs in promoter region and thus affecting their transcription. In this study, we determined LINC00460 as a hypoxia-inducible lncRNA. LINC00460 expression was upregulated under oxygen-deficient conditions and could be inhibited by knocking down HIF-1α. Two possible HREs in the promoter of the LINC00460 were calculated through the JASPAR database and confirmed that the HIF-1α binding site was HRE1. Additionally, we also found that inhibition of HIF-1α or LINC00460 inhibited the enhancement of cell proliferation and metastasis induced by hypoxia, and, remarkably, that overexpression of LINC00460 can partially reverse this inhibitory effect. These results show that LINC00460 is a hypoxia-induced lncRNA that participates in hypoxia-induced proliferation and metastasis in pancreatic cancer.

Through FISH experiments, we found that LINC00460 is located in the cytoplasm. Also, ceRNAs, crucial mechanisms for lncRNAs are located in the cytoplasm 41. Using prediction tools, we showed that LINC00460 may bind to miR-4689, and following RIP and dual-luciferase reporter assays confirmed this result. MiR-4689 plays an important role in inhibiting pancreatic cancer. Overexpression of MiR-4689 can partially reverse LINC00460 induced cell proliferation and metastasis. Collectively, LINC00460 plays the role of ceRNA by binding with miR-4689 in pancreatic cells. UBE2V1 was found to be upregulated in a variety of tumors. UBE2V1 was found to be the downstream target of miR-4689 and a dual-luciferase reporter assay was conducted to verify this. In addition, we identified that LINC00460 and UBE2V1 were significantly positively correlated in pancreatic cancer. Downregulation of UBE2V1 significantly inhibits the proliferation and metastasis of pancreatic cancer, while overexpression of LINC00460 can partially reverse the inhibitory effect of UBE2V1. Moreover, overexpression of LINC00460 led to upregulation of UBE2V1, while the overexpression of miR-4689 partially reversed the upregulation of UBE2V1. These results indicated that UBE2V1 plays a role of oncogene in pancreatic cancer and LINC00460 can further regulate UBE2V1 expression by regulating miR-4689. A previous study reported that UBE2V1 and its co-factor UBC13 could increase the stability of the p53 protein 42. Here, we found that downregulation of UBE2V1 decreased both wild-type and mutant p53 level. Although Co-IP assays indicated that UBE2V1 does not directly interact with p53, western blot showed that compared with the control group, the stability of wild type and mutant p53 in UBE2V1 knockdown group was significantly reduced. In brief, we showed that UBE2V1 mediates the stability of p53, and that knockdown of UBE2V1 decreased p53 protein levels in pancreatic cancer. Notably, knocking down UBE2V1 in wild-type p53 cells could promote tumor progression, implying that the biological functions of UBE2V1 in pancreatic cancer are p53 context-dependent. Thus, we demonstrated that the role of LINC00460 in pancreatic cancer is achieved by regulating its downstream molecules, miR-4689, and further mediates the expression of UBE2V1. UBE2V1 acts as an oncogene and stabilizes p53 in p53-mutant pancreatic cancer cells. In summary, LINC00460 promotes cell proliferation and metastasis by regulating the LINC00460/miR-4689/UBE2V1/p53 axis.

There is now evidence to support that USP10 plays diverse roles in different types of cancer 43. For example, USP10 could enhance the proliferation and metastasis of NSCLC by stabilizing HDAC7^44^, whereas USP10 can stabilize p53 and inhibit breast cancer progression 45. In the present study, we identified a relationship between LINC00460 and USP10 and found that USP10 may be a downstream target gene of LINC00460. RNA pull-down and RIP assays verified that USP10 and LINC00460 were directly combined. USP10 is highly expressed in pancreatic cancer cell lines and positively correlated with LINC00460. Downregulation of USP10 decreased the proliferative and metastatic ability of pancreatic cancer cells. The above results showed that USP10 promotes the development of pancreatic cancer by directly binding to LINC00460. USP10 was reported to deubiquitinate p53, increasing the stability of wild or mutant p53 protein 46. We found that USP10 binds directly to p53, and knocking down USP10 lowers p53 protein levels. Furthermore, immunofluorescence assays revealed that USP10 knockdown promoted the translocation of p53 out of the nucleus, which accelerates the degradation of p53 protein. Western blot assays indicated that knocking down USP10 significantly decreased the stability of p53. Interestingly, the downregulation of LINC00460 expression reduced the stability of p53 and the expression of USP10, and CoIP experiments revealed that the downregulation of LINC00460 partially hindered the mutual binding of USP10 and p53 in pancreatic cancer cells. This indicates that LINC00460 can mediate the expression of p53 by regulating USP10. A particularly important observation was that knockdown of USP10 in wild-type p53 cell lines promoted proliferation and metastasis, suggesting that USP10 could inhibited tumor development under the background of wild-type p53. This result is consistent with other studies (e.g., renal cell carcinoma 46) which reported that the biological function of USP10 depends on the p53 background, although the precise mechanism needs more in-depth study. In summary, we found that USP10 is a binding protein for LINC00460 and acts as an oncogene in mutated p53 pancreatic cancer cells. In addition, USP10 can regulate the translocation and stability of mutant p53, ultimately promoting the progression of pancreatic cancer.

Overall, we found that hypoxia-induced LINC00460 plays a role of oncogene in pancreatic cancer, and upregulation of LINC00460 is closely related to tumor proliferation, metastasis, and poor prognosis. Mechanistically, LINC00460 regulates its downstream target UBE2V1 through miR-4689, thereby increasing the stability of p53. In addition, LINC00460 promotes tumorigenesis by sequestering USP10, altering the stability and subcellular localization of p53. In short, hypoxia-induced LINC00460 enhances the stability of mutant p53 through the LINC00460/miR-4689/UBE2V1 axis and by sequestering USP10, and ultimately regulates pancreatic tumorigenesis. Our findings enriched the mechanism of lncRNAs in pancreatic cancer and identified LINC00460 as a possible biomarker for pancreatic cancer.

## Supplementary Material

Supplementary figures.

## Figures and Tables

**Figure 9:**
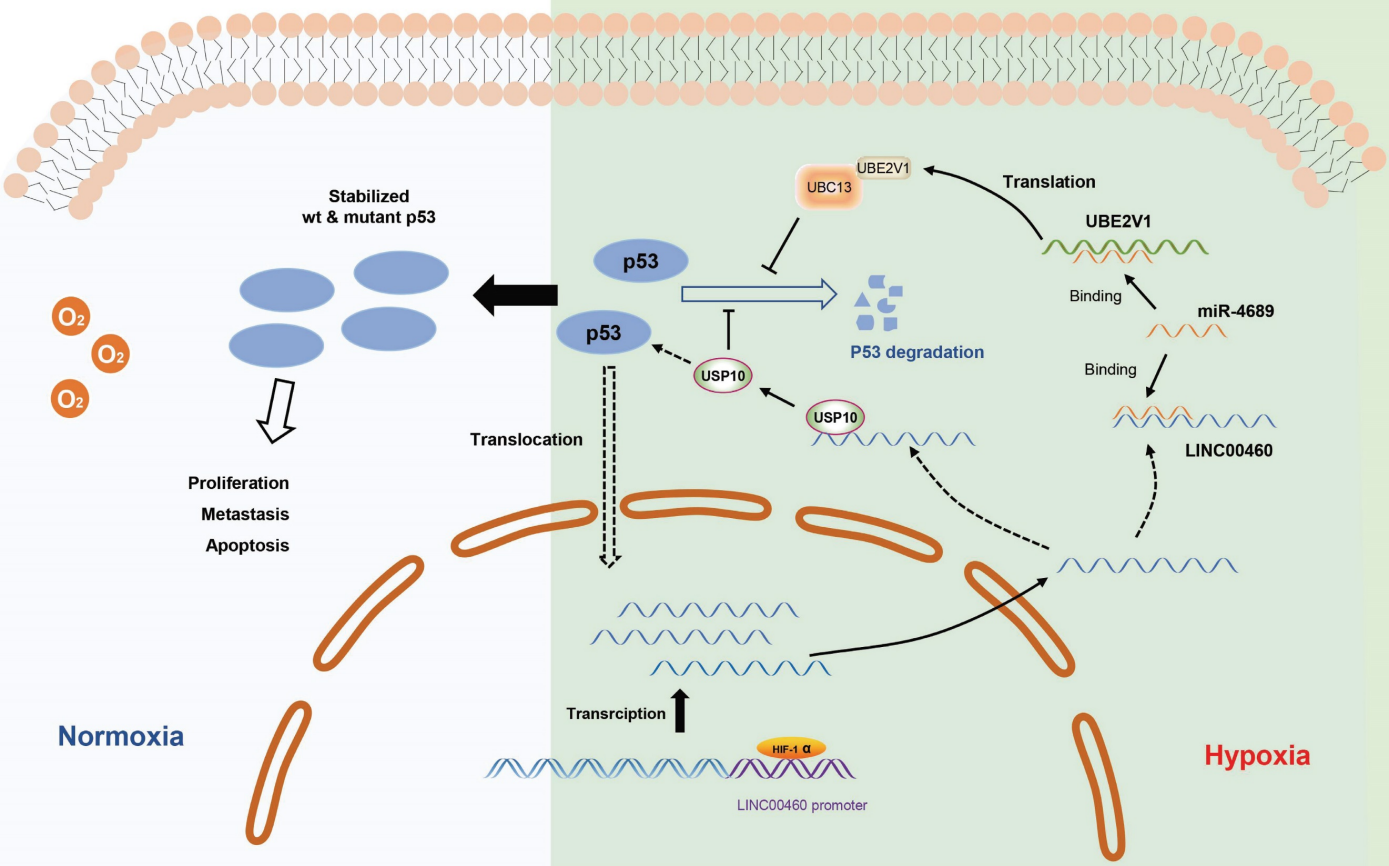
** The working model describing the upregulation and biological functions of LINC00460 in pancreatic cancer.** The upregulation of LINC00460 is induced by hypoxic environment of pancreatic cancer. LINC00460 promotes the progression of pancreatic cancer by regulating miR-4689/UBE2V1 axis and sequestering USP10; and ultimately enhance the stability of mutant p53 protein.

## References

[1] Siegel RL, Miller KD, Fuchs HE, Jemal A (2022). Cancer statistics, 2022. CA: a cancer journal for clinicians.

[2] Manoharan D, Chang LC, Wang LC, Shan YS, Lin FC, Wu LC (2021). Synchronization of Nanoparticle Sensitization and Radiosensitizing Chemotherapy through Cell Cycle Arrest Achieving Ultralow X-ray Dose Delivery to Pancreatic Tumors. ACS nano.

[3] Wood LD, Canto MI, Jaffee EM, Simeone DM (2022). Pancreatic Cancer: Pathogenesis, Screening, Diagnosis and Treatment. Gastroenterology.

[4] Chi Y, Wang D, Wang J, Yu W, Yang J (2019). Long Non-Coding RNA in the Pathogenesis of Cancers. Cells.

[5] Quinn JJ, Chang HY (2016). Unique features of long non-coding RNA biogenesis and function. Nature reviews Genetics.

[6] Sanchez Calle A, Kawamura Y, Yamamoto Y, Takeshita F, Ochiya T (2018). Emerging roles of long non-coding RNA in cancer. Cancer science.

[7] Hong H, Sui C, Qian T, Xu X, Zhu X, Fei Q (2020). Long noncoding RNA LINC00460 conduces to tumor growth and metastasis of hepatocellular carcinoma through miR-342-3p-dependent AGR2 up-regulation. Aging.

[8] Yang J, Lian Y, Yang R, Lian Y, Wu J, Liu J (2020). Upregulation of lncRNA LINC00460 Facilitates GC Progression through Epigenetically Silencing CCNG2 by EZH2/LSD1 and Indicates Poor Outcomes. Molecular therapy Nucleic acids.

[9] Cisneros-Villanueva M, Hidalgo-Pérez L, Cedro-Tanda A, Peña-Luna M, Mancera-Rodríguez MA, Hurtado-Cordova E (2021). LINC00460 Is a Dual Biomarker That Acts as a Predictor for Increased Prognosis in Basal-Like Breast Cancer and Potentially Regulates Immunogenic and Differentiation-Related Genes. Frontiers in oncology.

[10] Jiang Y, Cao W, Wu K, Qin X, Wang X, Li Y (2019). LncRNA LINC00460 promotes EMT in head and neck squamous cell carcinoma by facilitating peroxiredoxin-1 into the nucleus. Journal of experimental & clinical cancer research: CR.

[11] Li Y, Sun X (2022). An Effective Hypoxia-Related Long Non-Coding RNA Assessment Model for Prognosis of Lung Adenocarcinoma. Frontiers in genetics.

[12] Infantino V, Santarsiero A, Convertini P, Todisco S, Iacobazzi V (2021). Cancer Cell Metabolism in Hypoxia: Role of HIF-1 as Key Regulator and Therapeutic Target. International journal of molecular sciences.

[13] Wang R, Godet I, Yang Y, Salman S, Lu H, Lyu Y (2021). Hypoxia-inducible factor-dependent ADAM12 expression mediates breast cancer invasion and metastasis. Proceedings of the National Academy of Sciences of the United States of America.

[14] Tao J, Yang G, Zhou W, Qiu J, Chen G, Luo W (2021). Targeting hypoxic tumor microenvironment in pancreatic cancer. Journal of hematology & oncology.

[15] Chen P, He Z, Wang J, Xu J, Jiang X, Chen Y (2021). Hypoxia-Induced ZWINT Mediates Pancreatic Cancer Proliferation by Interacting With p53/p21. Frontiers in cell and developmental biology.

[16] Zhao J, Xiao A, Liu C, Ye C, Yin K, Lu M (2020). The HIF-1A/miR-17-5p/PDCD4 axis contributes to the tumor growth and metastasis of gastric cancer. Signal transduction and targeted therapy.

[17] Shi M, Dai WQ, Jia RR, Zhang QH, Wei J, Wang YG (2021). APC(CDC20)-mediated degradation of PHD3 stabilizes HIF-1a and promotes tumorigenesis in hepatocellular carcinoma. Cancer letters.

[18] Deng SJ, Chen HY, Ye Z, Deng SC, Zhu S, Zeng Z (2018). Hypoxia-induced LncRNA-BX111 promotes metastasis and progression of pancreatic cancer through regulating ZEB1 transcription. Oncogene.

[19] Hu G, Ma J, Zhang J, Chen Y, Liu H, Huang Y (2021). Hypoxia-induced lncHILAR promotes renal cancer metastasis via ceRNA for the miR-613/206/ 1-1-3p/Jagged-1/Notch/CXCR4 signaling pathway. Molecular therapy: the journal of the American Society of Gene Therapy.

[20] Ho KH, Shih CM, Liu AJ, Chen KC (2022). Hypoxia-inducible lncRNA MIR210HG interacting with OCT1 is involved in glioblastoma multiforme malignancy. Cancer science.

[21] Hiraki M, Nishimura J, Takahashi H, Wu X, Takahashi Y, Miyo M (2015). Concurrent Targeting of KRAS and AKT by MiR-4689 Is a Novel Treatment Against Mutant KRAS Colorectal Cancer. Molecular therapy Nucleic acids.

[22] Xu N, Gulick J, Osinska H, Yu Y, McLendon PM, Shay-Winkler K (2020). Ube2v1 Positively Regulates Protein Aggregation by Modulating Ubiquitin Proteasome System Performance Partially Through K63 Ubiquitination. Circulation research.

[23] Shen T, Cai LD, Liu YH, Li S, Gan WJ, Li XM (2018). Ube2v1-mediated ubiquitination and degradation of Sirt1 promotes metastasis of colorectal cancer by epigenetically suppressing autophagy. Journal of hematology & oncology.

[24] Dikshit A, Jin YJ, Degan S, Hwang J, Foster MW, Li CY (2018). UBE2N Promotes Melanoma Growth via MEK/FRA1/SOX10 Signaling. Cancer research.

[25] Wang X, Xia S, Li H, Wang X, Li C, Chao Y (2020). The deubiquitinase USP10 regulates KLF4 stability and suppresses lung tumorigenesis. Cell death and differentiation.

[26] Li B, Qi ZP, He DL, Chen ZH, Liu JY, Wong MW (2021). NLRP7 deubiquitination by USP10 promotes tumor progression and tumor-associated macrophage polarization in colorectal cancer. Journal of experimental & clinical cancer research: CR.

[27] Gao D, Zhang Z, Xu R, He Z, Li F, Hu Y (2022). The Prognostic Value and Immune Infiltration of USP10 in Pan-Cancer: A Potential Therapeutic Target. Frontiers in oncology.

[28] Chen L, Liu S, Tao Y (2020). Regulating tumor suppressor genes: post-translational modifications. Signal transduction and targeted therapy.

[29] Muller PA, Vousden KH (2013). p53 mutations in cancer. Nature cell biology.

[30] Oren M, Rotter V (2010). Mutant p53 gain-of-function in cancer. Cold Spring Harbor perspectives in biology.

[31] Zhang C, Liu J, Xu D, Zhang T, Hu W, Feng Z (2020). Gain-of-function mutant p53 in cancer progression and therapy. Journal of molecular cell biology.

[32] Baslan T, Morris JPt, Zhao Z, Reyes J, Ho YJ, Tsanov KM (2022). Ordered and deterministic cancer genome evolution after p53 loss. Nature.

[33] Mukherjee S, Maddalena M, Lü Y, Martinez S, Nataraj NB, Noronha A (2022). Cross-talk between mutant p53 and p62/SQSTM1 augments cancer cell migration by promoting the degradation of cell adhesion proteins. Proceedings of the National Academy of Sciences of the United States of America.

[34] Laine A, Topisirovic I, Zhai D, Reed JC, Borden KL, Ronai Z (2006). Regulation of p53 localization and activity by Ubc13. Molecular and cellular biology.

[35] Kwon SK, Saindane M, Baek KH (2017). p53 stability is regulated by diverse deubiquitinating enzymes. Biochimica et biophysica acta Reviews on cancer.

[36] Li Y, Hu J, Guo D, Ma W, Zhang X, Zhang Z (2022). LncRNA SNHG5 promotes the proliferation and cancer stem cell-like properties of HCC by regulating UPF1 and Wnt-signaling pathway. Cancer gene therapy.

[37] Huang G, Xiang Z, Wu H, He Q, Dou R, Lin Z (2022). The lncRNA BDNF-AS/WDR5/FBXW7 axis mediates ferroptosis in gastric cancer peritoneal metastasis by regulating VDAC3 ubiquitination. International journal of biological sciences.

[38] Zhao C, Ling X, Xia Y, Yan B, Guan Q (2022). LncRNA UCA1 promotes SOX12 expression in breast cancer by regulating m(6)A modification of miR-375 by METTL14 through DNA methylation. Cancer gene therapy.

[40] Xing Z, Li S, Xing J, Yu G, Wang G, Liu Z (2022). Silencing of LINC01963 enhances the chemosensitivity of prostate cancer cells to docetaxel by targeting the miR-216b-5p/TrkB axis. Laboratory investigation; a journal of technical methods and pathology.

[41] Tay Y, Rinn J, Pandolfi PP (2014). The multilayered complexity of ceRNA crosstalk and competition. Nature.

[42] Cheng J, Fan YH, Xu X, Zhang H, Dou J, Tang Y (2014). A small-molecule inhibitor of UBE2N induces neuroblastoma cell death via activation of p53 and JNK pathways. Cell death & disease.

[43] Tao L, Liu X, Jiang X, Zhang K, Wang Y, Li X (2022). USP10 as a Potential Therapeutic Target in Human Cancers. Genes.

[44] Guo K, Ma Z, Zhang Y, Han L, Shao C, Feng Y (2022). HDAC7 promotes NSCLC proliferation and metastasis via stabilization by deubiquitinase USP10 and activation of β-catenin-FGF18 pathway. Journal of experimental & clinical cancer research: CR.

[45] Yang R, Chen H, Xing L, Wang B, Hu M, Ou X (2022). Hypoxia-induced circWSB1 promotes breast cancer progression through destabilizing p53 by interacting with USP10. Molecular cancer.

[46] Yuan J, Luo K, Zhang L, Cheville JC, Lou Z (2010). USP10 regulates p53 localization and stability by deubiquitinating p53. Cell.

