# Supplementary material for: Hypoxia-induced long non-coding RNA LINC00460 promotes p53 mediated proliferation and metastasis of pancreatic cancer by regulating the miR-4689/UBE2V1 axis and sequestering USP10
Source: Int J Med Sci. 2023 Sep 4;20(10):1339–57. doi: 10.7150/ijms.87833 (PMC10542025; doi:10.7150/ijms.87833)
Supplement: Supplementary file 1 — Supplementary figures. [file ijmsv20p1339s1.pdf]

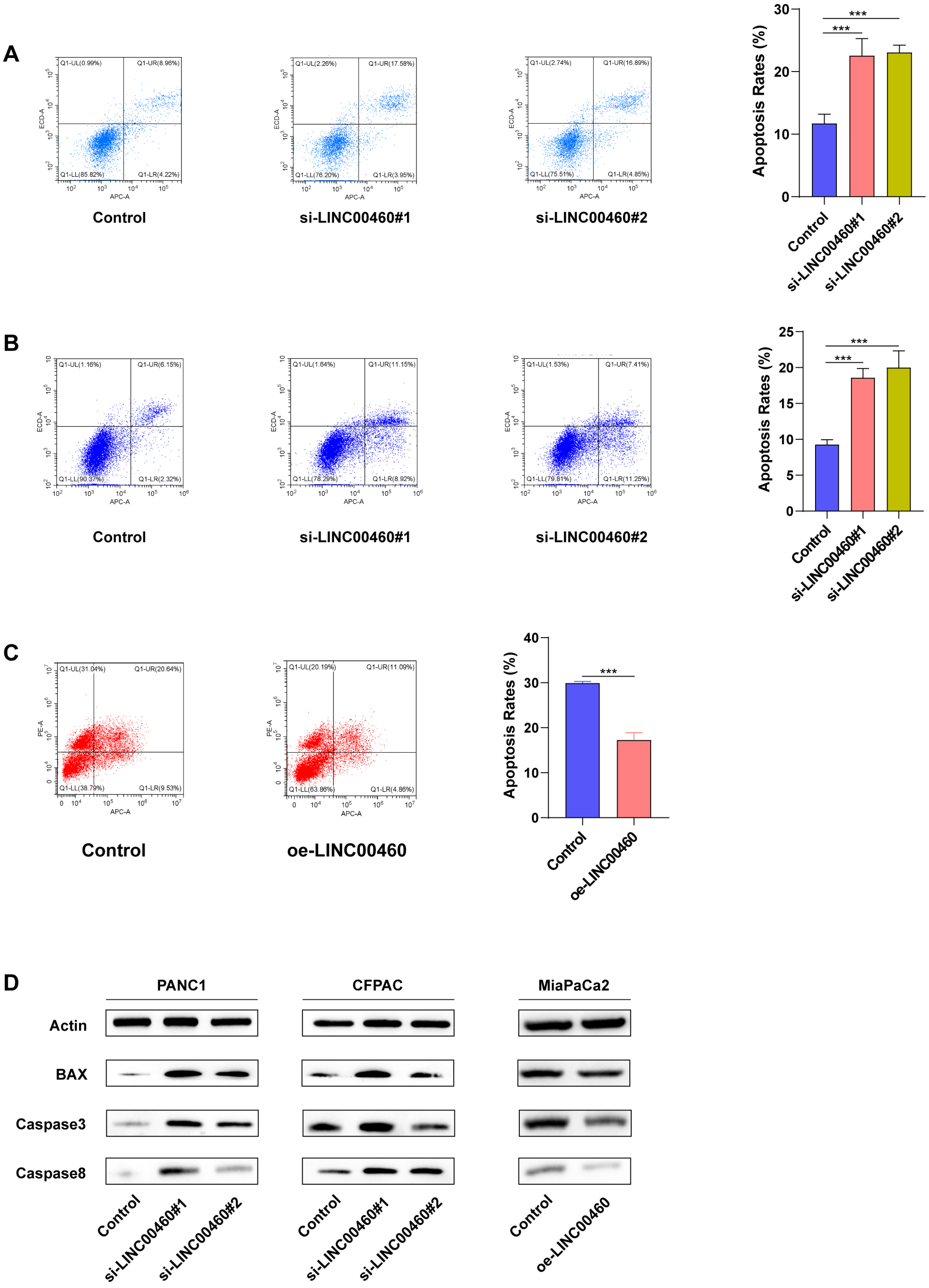
A
Q1-UL(0.99%)
Q1-UR(8.96%)
Q1-LL(85.82%)
Q1-LR(4.22%)
Control
Q1-UL(2.26%)
Q1-UR(17.58%)
Q1-LL(76.20%)
Q1-LR(3.95%)
si-LINC00460#1
Q1-UL(2.74%)
Q1-UR(16.89%)
Q1-LL(75.51%)
Q1-LR(4.85%)
si-LINC00460#2
ECD-A
APC-A
Apoptosis Rates (%)
***
***
Control
si-LINC00460#1
si-LINC00460#2
B
Q1-UL(1.16%)
Q1-UR(6.15%)
Q1-LL(90.37%)
Q1-LR(2.32%)
Control
Q1-UL(1.64%)
Q1-UR(11.15%)
Q1-LL(78.29%)
Q1-LR(8.92%)
si-LINC00460#1
Q1-UL(1.53%)
Q1-UR(7.41%)
Q1-LL(79.81%)
Q1-LR(11.25%)
si-LINC00460#2
Apoptosis Rates (%)
***
***
C
Q1-UL(31.04%)
Q1-UR(20.64%)
Q1-LL(38.79%)
Q1-LR(9.53%)
Control
Q1-UL(20.19%)
Q1-UR(11.09%)
Q1-LL(63.86%)
Q1-LR(4.86%)
oe-LINC00460
PE-A
APC-A
Apoptosis Rates (%)
***
Control
oe-LINC00460
D
PANC1
CFPAC
MiaPaCa2
Actin
BAX
Caspase3
Caspase8
Control
si-LINC00460#1
si-LINC00460#2
Control
si-LINC00460#1
si-LINC00460#2
Control
oe-LINC00460

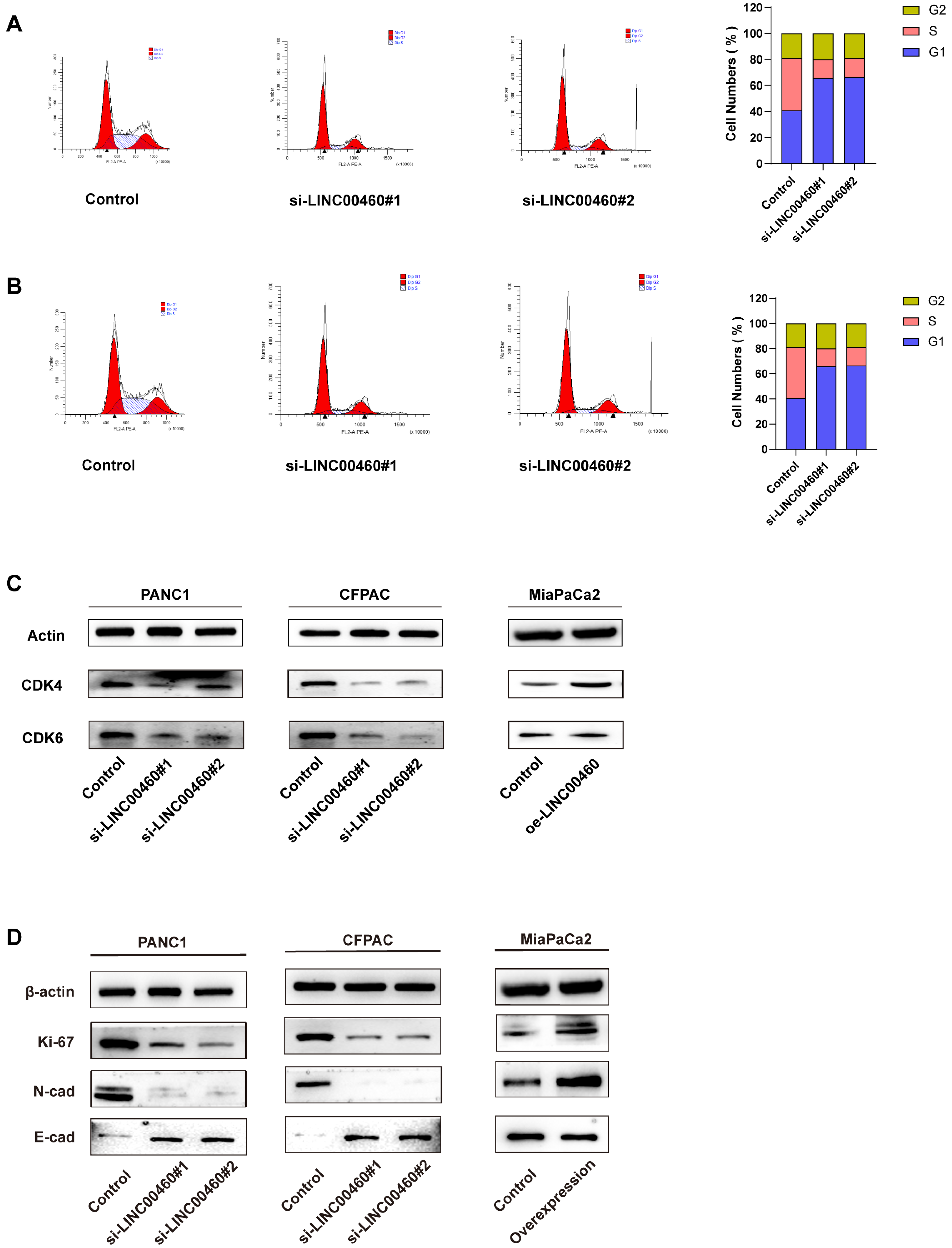

A
Control
si-LINC00460#1
si-LINC00460#2
Cell Numbers (%)
G2
S
G1
B
C
PANC1
CFPAC
MiaPaCa2
Actin
CDK4
CDK6
Control
si-LINC00460#1
si-LINC00460#2
oe-LINC00460
D
β-actin
Ki-67
N-cad
E-cad
Overexpression

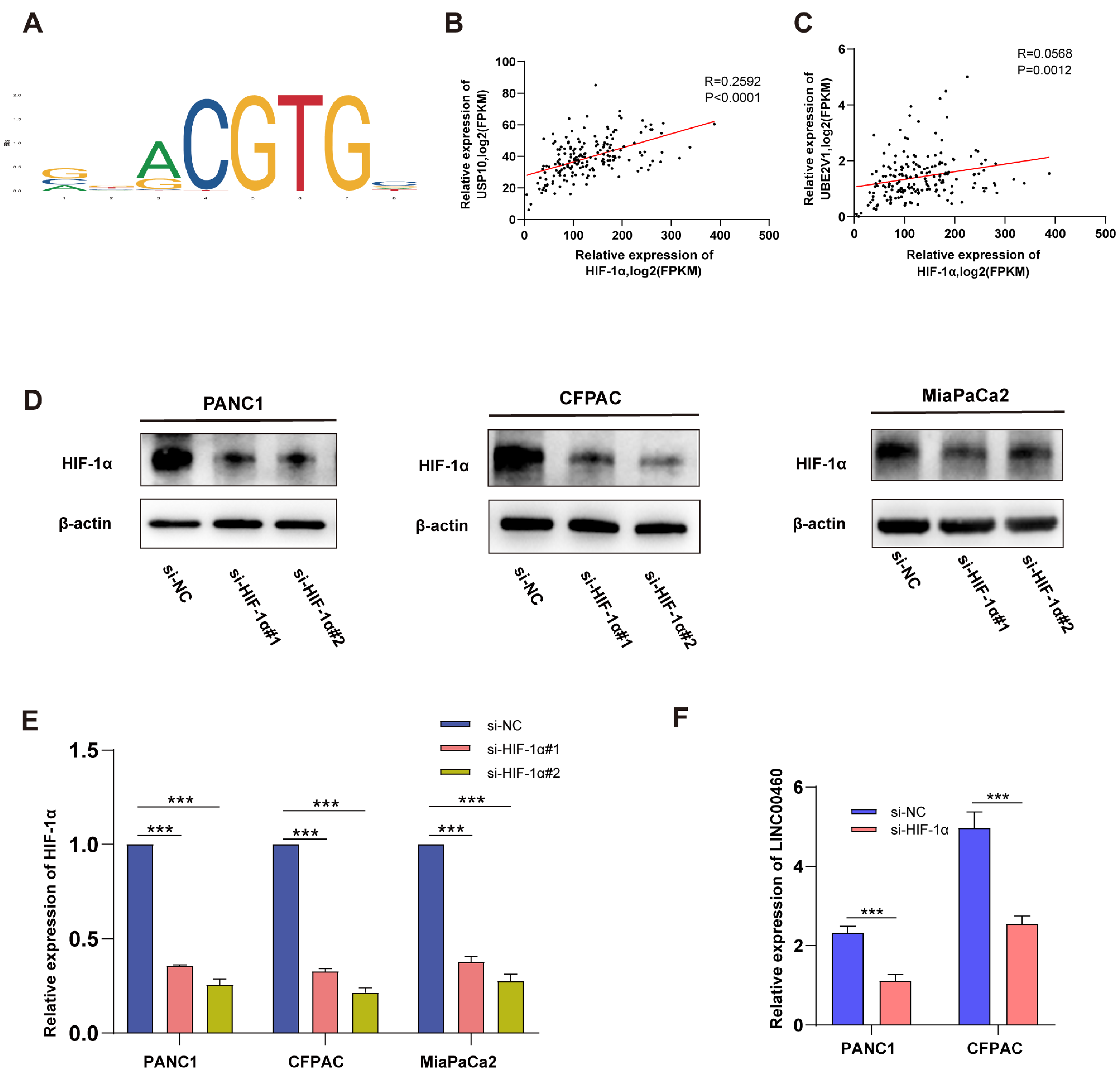
A
B
R=0.2592
P<0.0001
Relative expression of USP10,log2(FPKM)
Relative expression of HIF-1α,log2(FPKM)
C
R=0.0568
P=0.0012
Relative expression of UBE2V1,log2(FPKM)
Relative expression of HIF-1α,log2(FPKM)
D
PANC1
CFPAC
MiaPaCa2
HIF-1α
β-actin
si-NC
si-HIF-1α#1
si-HIF-1α#2
E
si-NC
si-HIF-1α#1
si-HIF-1α#2
Relative expression of HIF-1α
***
PANC1
CFPAC
MiaPaCa2
F
si-NC
si-HIF-1α
Relative expression of LINC00460
***
PANC1
CFPAC

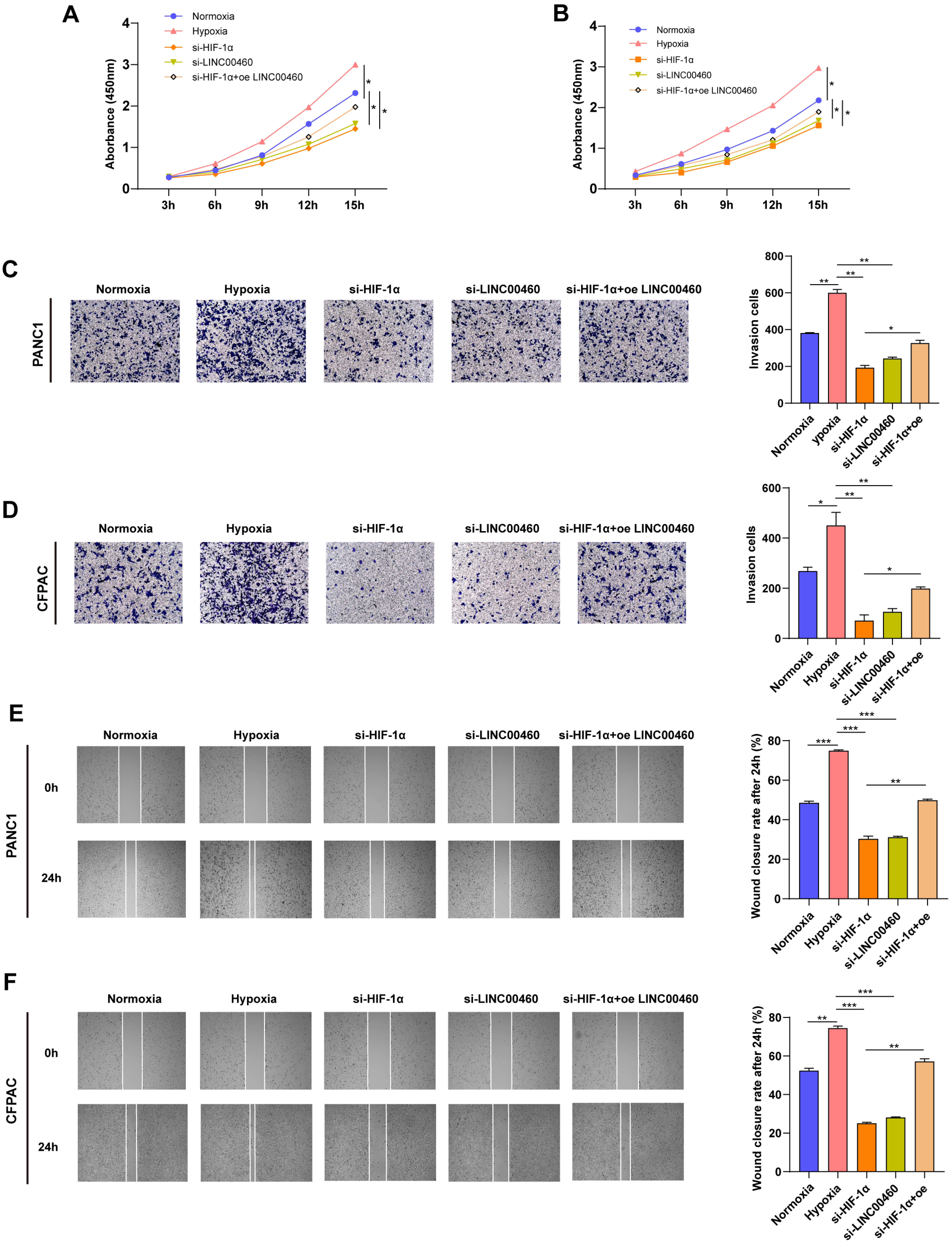
A
Normoxia
Hypoxia
si-HIF-1α
si-LINC00460
si-HIF-1α+oe LINC00460
Aborbance (450nm)
3h
6h
9h
12h
15h
B
C
PANC1
Invasion cells
D
CFPAC
E
0h
24h
Wound closure rate after 24h (%)
F
si-HIF-1α+oe
ypoxia

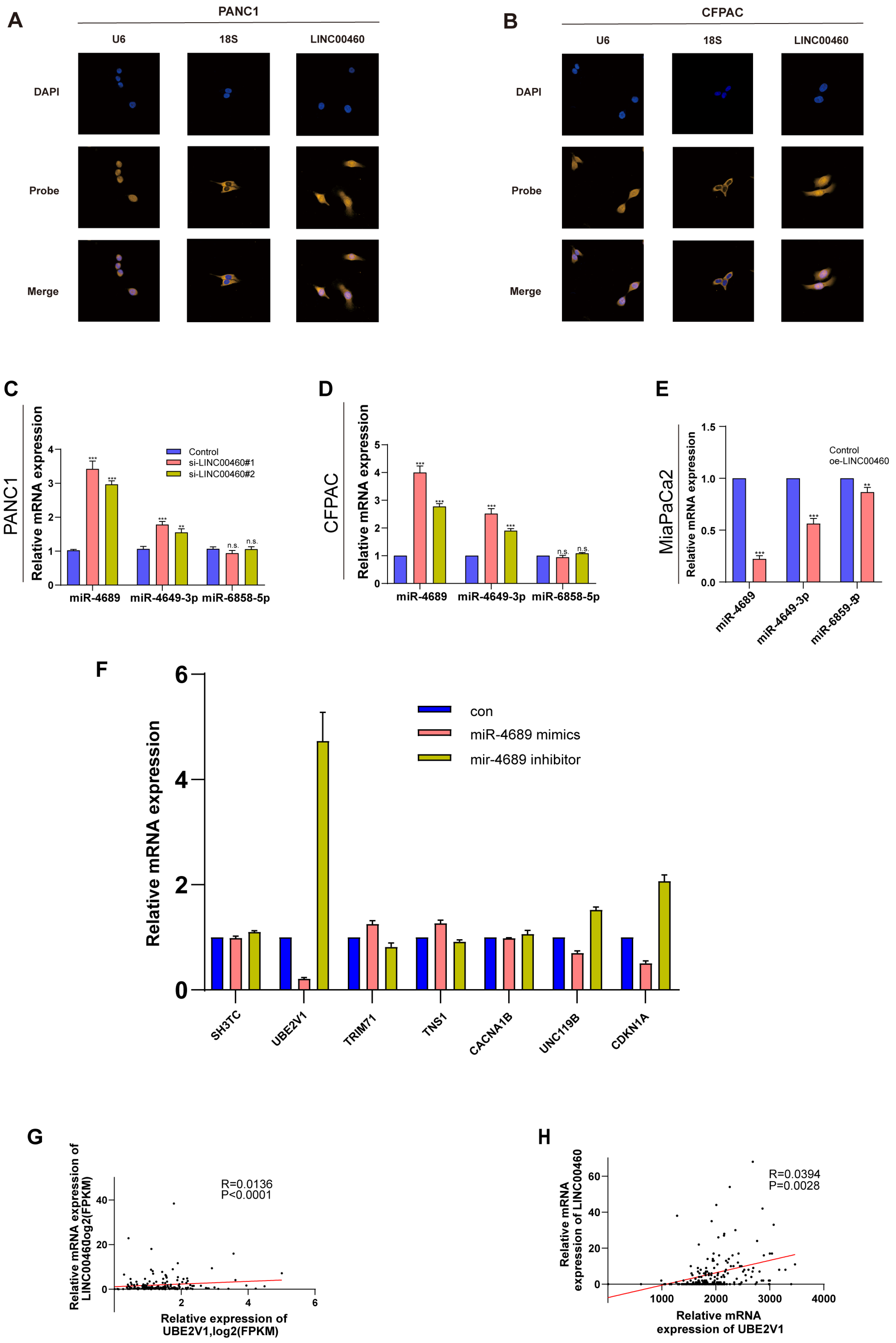
A
PANC1
U6
18S
LINC00460
DAPI
Probe
Merge
B
CFPAC
U6
18S
LINC00460
DAPI
Probe
Merge
C
PANC1
Relative mRNA expression
Control
si-LINC00460#1
si-LINC00460#2
***
**
n.s.
miR-4689
miR-4649-3p
miR-6858-5p
D
CFPAC
Relative mRNA expression
miR-4689
miR-4649-3p
miR-6858-5p
E
MiaPaCa2
Relative mRNA expression
Control
oe-LINC00460
miR-4689
miR-4649-3p
miR-6859-5p
F
Relative mRNA expression
con
miR-4689 mimics
mir-4689 inhibitor
SH3TC
UBE2V1
TRIM71
TNS1
CACNA1B
UNC119B
CDKN1A
G
Relative mRNA expression of LINC00460log2(FPKM)
R=0.0136
P<0.0001
Relative expression of UBE2V1,log2(FPKM)
H
Relative mRNA expression of LINC00460
R=0.0394
P=0.0028
Relative mRNA expression of UBE2V1

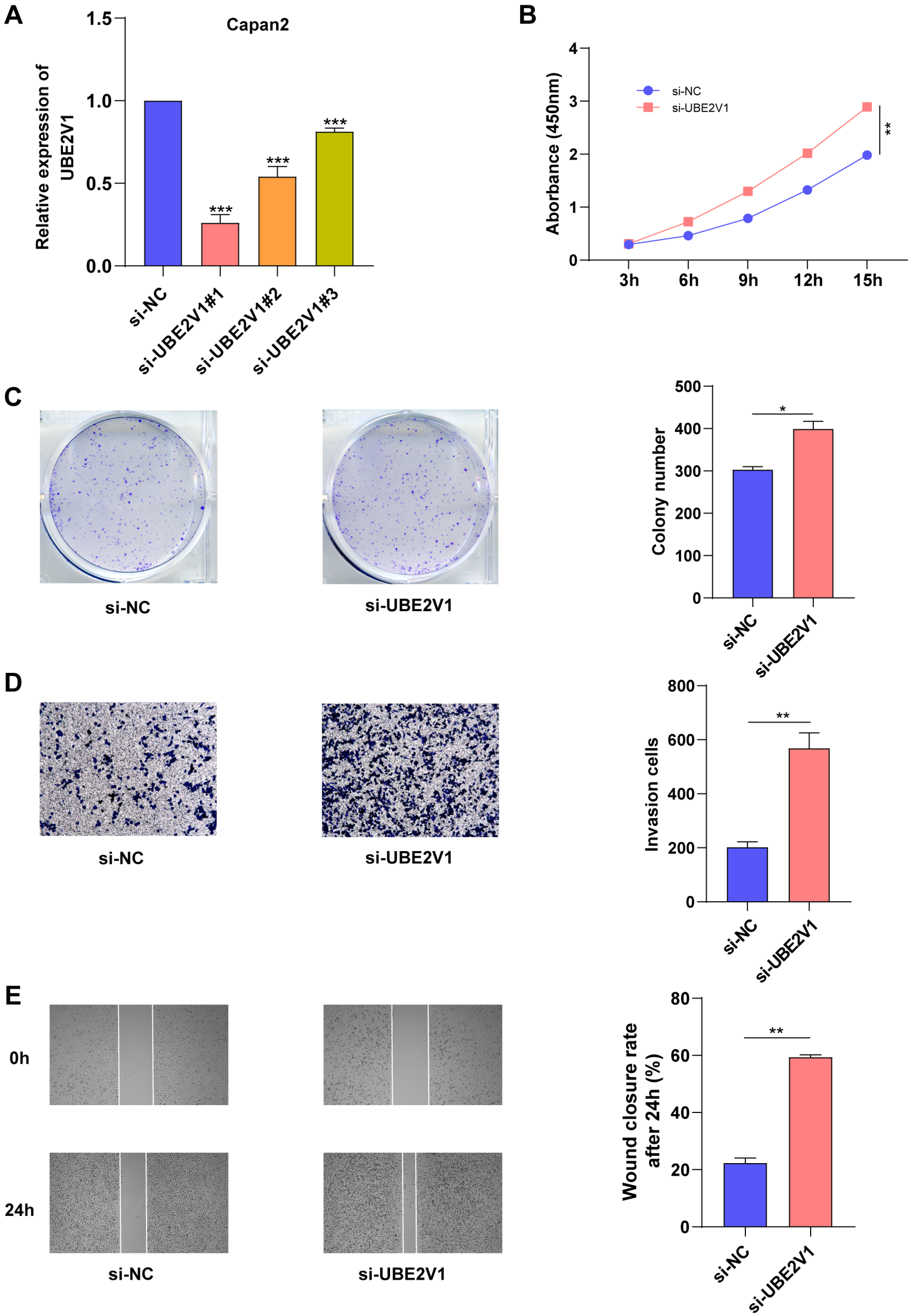
A
Capan2
Relative expression of UBE2V1
1.5
1.0
0.5
0.0
***
***
***
si-NC
si-UBE2V1#1
si-UBE2V1#2
si-UBE2V1#3
B
Aborbance (450nm)
si-NC
si-UBE2V1
**
3h
6h
9h
12h
15h
C
si-NC
si-UBE2V1
Colony number
*
D
si-NC
si-UBE2V1
Invasion cells
**
E
0h
24h
si-NC
si-UBE2V1
Wound closure rate after 24h (%)
**

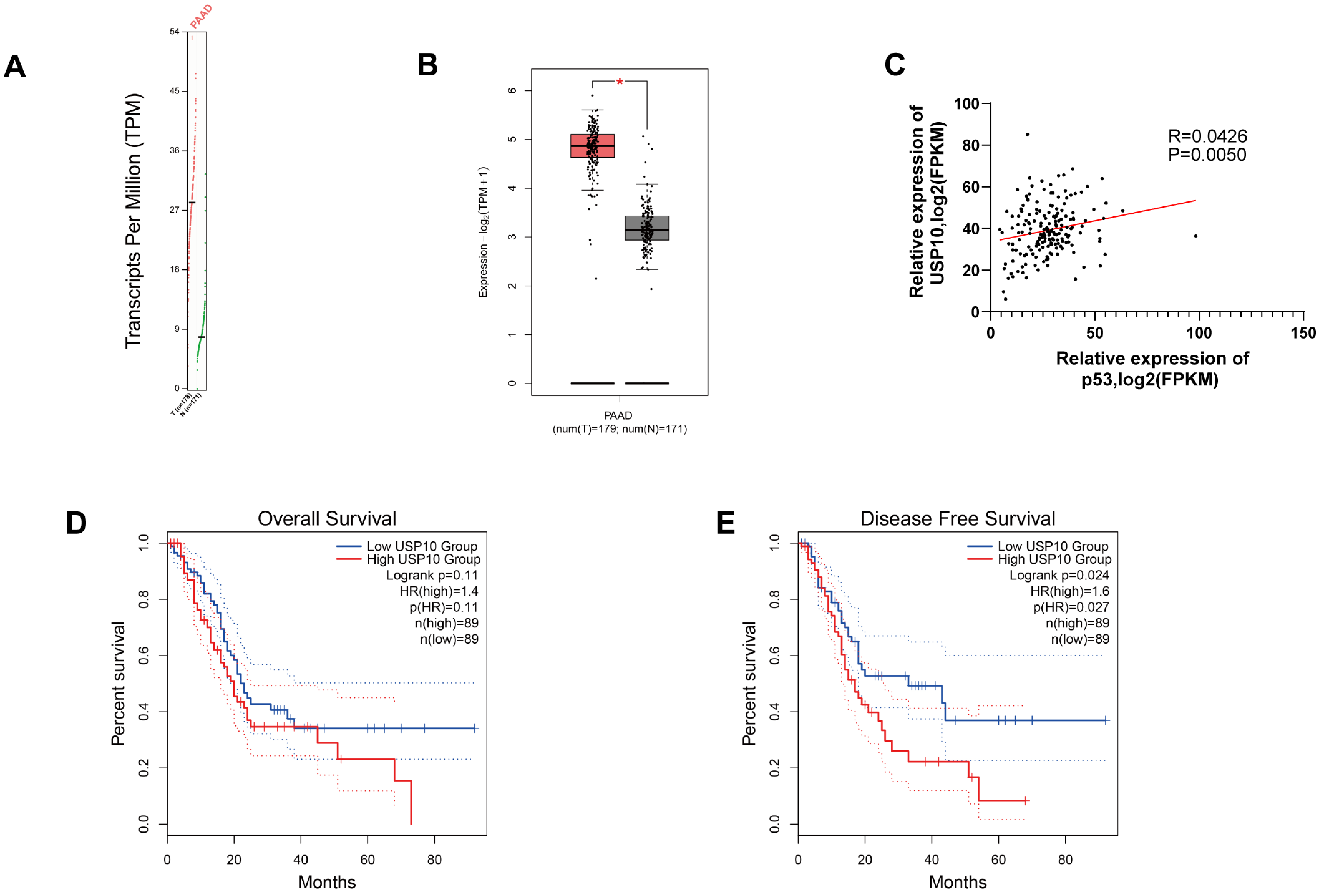
A
PAAD
Transcripts Per Million (TPM)
T (n=178)
N (n=171)
B
Expression − log2(TPM + 1)
*
PAAD
(num(T)=179; num(N)=171)
C
R=0.0426
P=0.0050
Relative expression of USP10,log2(FPKM)
Relative expression of p53,log2(FPKM)
D
Overall Survival
Low USP10 Group
High USP10 Group
Logrank p=0.11
HR(high)=1.4
p(HR)=0.11
n(high)=89
n(low)=89
Percent survival
Months
E
Disease Free Survival
Low USP10 Group
High USP10 Group
Logrank p=0.024
HR(high)=1.6
p(HR)=0.027
n(high)=89
n(low)=89
Percent survival
Months

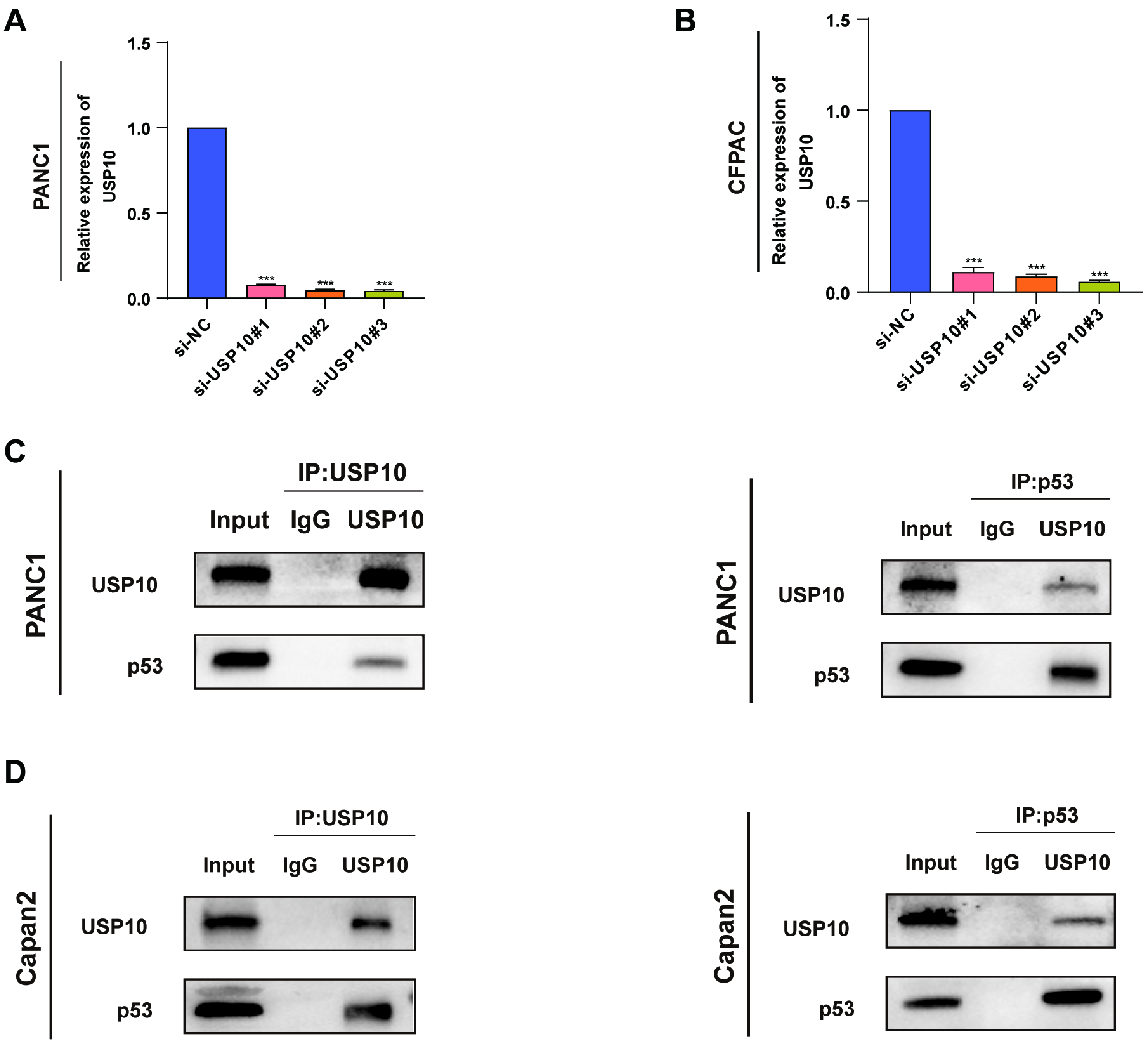

A
PANC1
Relative expression of USP10
1.5
1.0
0.5
0.0
***
si-NC
si-USP10#1
si-USP10#2
si-USP10#3
B
CFPAC
Relative expression of USP10
1.5
1.0
0.5
0.0
***
si-NC
si-USP10#1
si-USP10#2
si-USP10#3
C
PANC1
IP:USP10
Input
IgG
USP10
USP10
p53
PANC1
IP:p53
Input
IgG
USP10
USP10
p53
D
Capan2
IP:USP10
Input
IgG
USP10
USP10
p53
Capan2
IP:p53
Input
IgG
USP10
USP10
p53

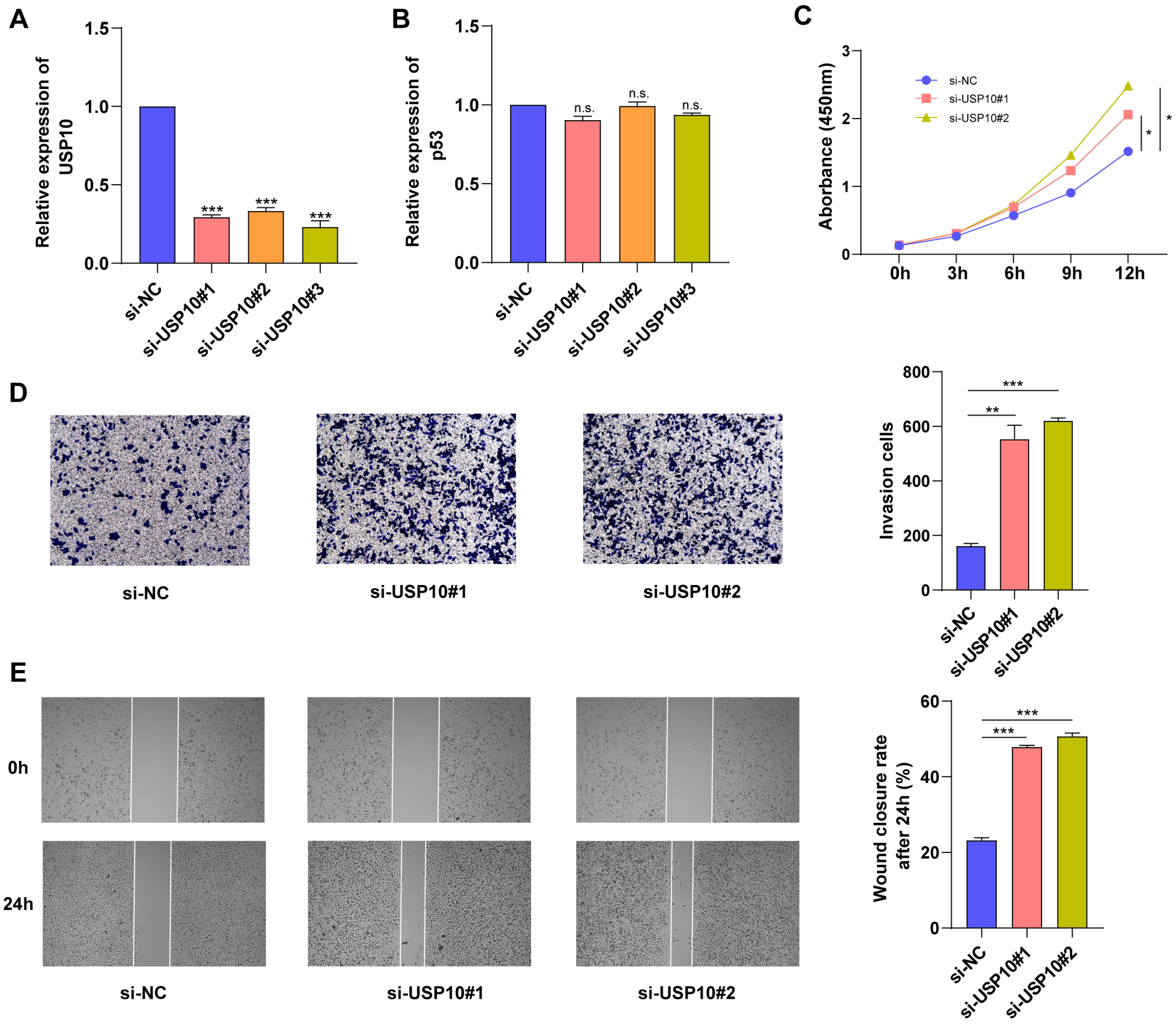
A
Relative expression of USP10
1.5
1.0
0.5
0.0
***
***
***
si-NC
si-USP10#1
si-USP10#2
si-USP10#3
B
Relative expression of p53
n.s.
n.s.
n.s.
C
Aborbance (450nm)
si-NC
si-USP10#1
si-USP10#2
*
*
0h
3h
6h
9h
12h
D
si-NC
si-USP10#1
si-USP10#2
Invasion cells
800
600
400
200
0
**
***
E
0h
24h
Wound closure rate after 24h (%)
60
40
20
0
***
***

A

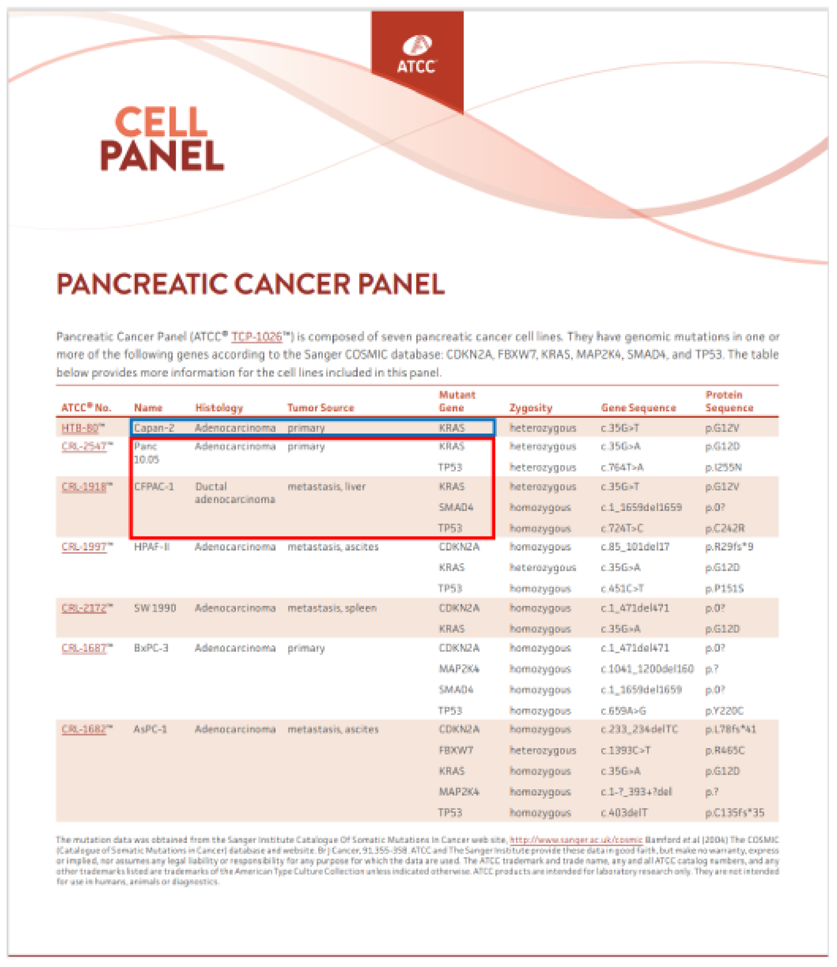

ATCC

CELL PANEL

PANCREATIC CANCER PANEL

Pancreatic Cancer Panel (ATCC® TCP-1026™) is composed of seven pancreatic cancer cell lines. They have genomic mutations in one or more of the following genes according to the Sanger COSMIC database: CDKN2A, FBXW7, KRAS, MAP2K4, SMAD4, and TP53. The table below provides more information for the cell lines included in this panel.

ATCC® No.	Name	Histology	Tumor Source	Mutant Gene	Zygosity	Gene Sequence	Protein Sequence
HTB-80™	Capan-2	Adenocarcinoma	primary	KRAS	heterozygous	c.35G>T	p.G12V
CRL-2547™	Panc 10.05	Adenocarcinoma	primary	KRAS	heterozygous	c.35G>A	p.G12D
				TP53	heterozygous	c.764T>A	p.I255N
CRL-1918™	CFPAC-1	Ductal adenocarcinoma	metastasis, liver	KRAS	heterozygous	c.35G>T	p.G12V
				SMAD4	homozygous	c.1_1659del1659	p.0?
				TP53	homozygous	c.724T>C	p.C242R
CRL-1997™	HPAF-II	Adenocarcinoma	metastasis, ascites	CDKN2A	homozygous	c.85_101del17	p.R29fs*9
				KRAS	heterozygous	c.35G>A	p.G12D
				TP53	homozygous	c.451C>T	p.P151S
CRL-2172™	SW 1990	Adenocarcinoma	metastasis, spleen	CDKN2A	homozygous	c.1_471del471	p.0?
				KRAS	homozygous	c.35G>A	p.G12D
CRL-1687™	BxPC-3	Adenocarcinoma	primary	CDKN2A	homozygous	c.1_471del471	p.0?
				MAP2K4	homozygous	c.1041_1200del160	p.?
				SMAD4	homozygous	c.1_1659del1659	p.0?
				TP53	homozygous	c.659A>G	p.Y220C
CRL-1682™	AsPC-1	Adenocarcinoma	metastasis, ascites	CDKN2A	homozygous	c.233_234delTC	p.L78fs*41
				FBXW7	heterozygous	c.1393C>T	p.R465C
				KRAS	homozygous	c.35G>A	p.G12D
				MAP2K4	homozygous	c.1-?_393+?del	p.?
				TP53	homozygous	c.403delT	p.C135fs*35

The mutation data was obtained from the Sanger Institute Catalogue Of Somatic Mutations In Cancer web site, http://www.sanger.ac.uk/cosmic Bamford et al (2004) The COSMIC (Catalogue of Somatic Mutations in Cancer) database and website. Br J Cancer, 91,355-358. ATCC and The Sanger Institute provide these data in good faith, but make no warranty, express or implied, nor assumes any legal liability or responsibility for any purpose for which the data are used. The ATCC trademark and trade name, any and all ATCC catalog numbers, and any other trademarks listed are trademarks of the American Type Culture Collection unless indicated otherwise. ATCC products are intended for laboratory research only. They are not intended for use in humans, animals or diagnostics.

B

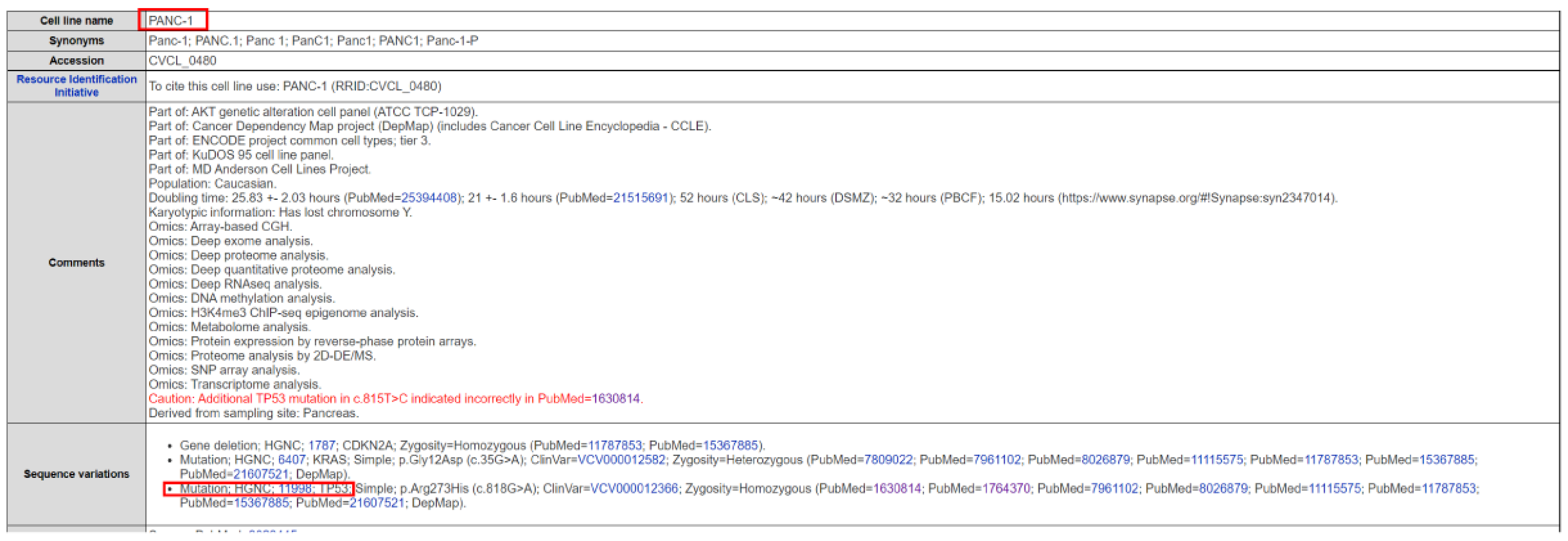

Cell line name	PANC-1
Synonyms	Panc-1; PANC.1; Panc 1; PanC1; Panc1; PANC1; Panc-1-P
Accession	CVCL_0480
Resource Identification Initiative	To cite this cell line use: PANC-1 (RRID:CVCL_0480)
Comments	Part of: AKT genetic alteration cell panel (ATCC TCP-1029).<br>Part of: Cancer Dependency Map project (DepMap) (includes Cancer Cell Line Encyclopedia - CCLE).<br>Part of: ENCODE project common cell types; tier 3.<br>Part of: KuDOS 95 cell line panel.<br>Part of: MD Anderson Cell Lines Project.<br>Population: Caucasian.<br>Doubling time: 25.83 +- 2.03 hours (PubMed=25394408); 21 +- 1.6 hours (PubMed=21515691); 52 hours (CLS); ~42 hours (DSMZ); ~32 hours (PBCF); 15.02 hours (https://www.synapse.org/#!Synapse:syn2347014).<br>Karyotypic information: Has lost chromosome Y.<br>Omics: Array-based CGH.<br>Omics: Deep exome analysis.<br>Omics: Deep proteome analysis.<br>Omics: Deep quantitative proteome analysis.<br>Omics: Deep RNAseq analysis.<br>Omics: DNA methylation analysis.<br>Omics: H3K4me3 ChIP-seq epigenome analysis.<br>Omics: Metabolome analysis.<br>Omics: Protein expression by reverse-phase protein arrays.<br>Omics: Proteome analysis by 2D-DE/MS.<br>Omics: SNP array analysis.<br>Omics: Transcriptome analysis.<br>Caution: Additional TP53 mutation in c.815T>C indicated incorrectly in PubMed=1630814.<br>Derived from sampling site: Pancreas.
Sequence variations	• Gene deletion; HGNC; 1787; CDKN2A; Zygosity=Homozygous (PubMed=11787853; PubMed=15367885).<br>• Mutation; HGNC; 6407; KRAS; Simple; p.Gly12Asp (c.35G>A); ClinVar=VCV000012582; Zygosity=Heterozygous (PubMed=7809022; PubMed=7961102; PubMed=8026879; PubMed=11115575; PubMed=11787853; PubMed=15367885; PubMed=21607521; DepMap).<br>• Mutation; HGNC; 11998; TP53; Simple; p.Arg273His (c.818G>A); ClinVar=VCV000012366; Zygosity=Homozygous (PubMed=1630814; PubMed=1764370; PubMed=7961102; PubMed=8026879; PubMed=11115575; PubMed=11787853; PubMed=15367885; PubMed=21607521; DepMap).

C

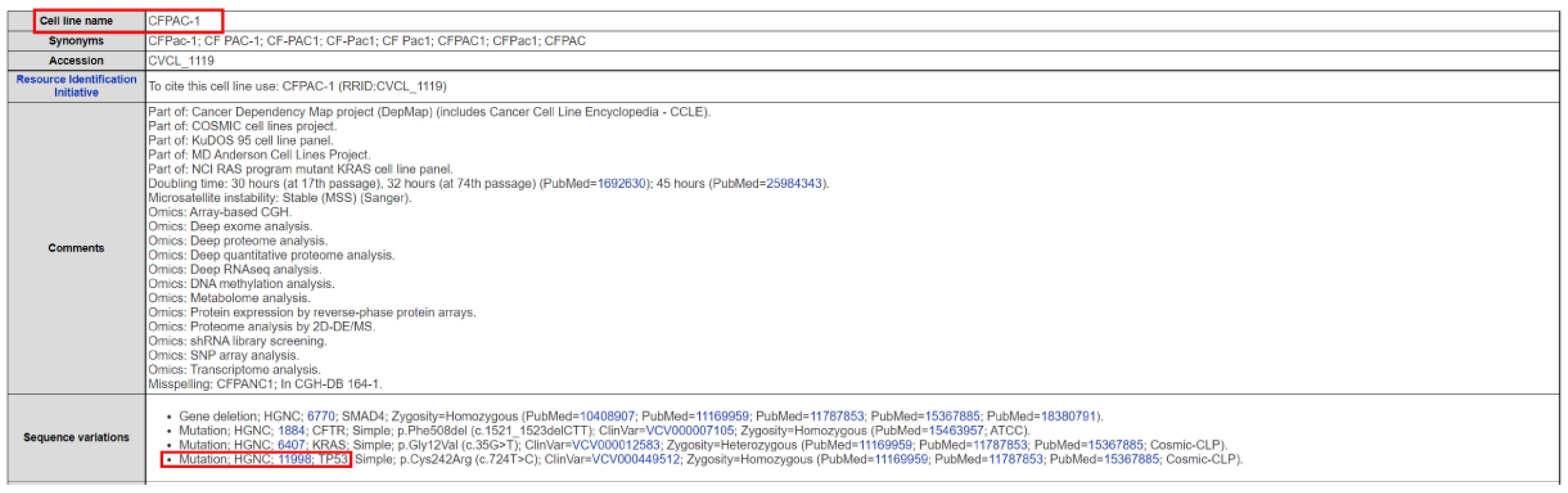

Cell line name	CFPAC-1
Synonyms	CFPac-1; CF PAC-1; CF-PAC1; CF-Pac1; CF Pac1; CFPAC1; CFPac1; CFPAC
Accession	CVCL_1119
Resource Identification Initiative	To cite this cell line use: CFPAC-1 (RRID:CVCL_1119)
Comments	Part of: Cancer Dependency Map project (DepMap) (includes Cancer Cell Line Encyclopedia - CCLE).<br>Part of: COSMIC cell lines project.<br>Part of: KuDOS 95 cell line panel.<br>Part of: MD Anderson Cell Lines Project.<br>Part of: NCI RAS program mutant KRAS cell line panel.<br>Doubling time: 30 hours (at 17th passage), 32 hours (at 74th passage) (PubMed=1692630); 45 hours (PubMed=25984343).<br>Microsatellite instability: Stable (MSS) (Sanger).<br>Omics: Array-based CGH.<br>Omics: Deep exome analysis.<br>Omics: Deep proteome analysis.<br>Omics: Deep quantitative proteome analysis.<br>Omics: Deep RNAseq analysis.<br>Omics: DNA methylation analysis.<br>Omics: Metabolome analysis.<br>Omics: Protein expression by reverse-phase protein arrays.<br>Omics: Proteome analysis by 2D-DE/MS.<br>Omics: shRNA library screening.<br>Omics: SNP array analysis.<br>Omics: Transcriptome analysis.<br>Misspelling: CFPANC1; In CGH-DB 164-1.
Sequence variations	• Gene deletion; HGNC; 6770; SMAD4; Zygosity=Homozygous (PubMed=10408907; PubMed=11169959; PubMed=11787853; PubMed=15367885; PubMed=18380791).<br>• Mutation; HGNC; 1884; CFTR; Simple; p.Phe508del (c.1521_1523delCTT); ClinVar=VCV000007105; Zygosity=Homozygous (PubMed=15463957; ATCC).<br>• Mutation; HGNC; 6407; KRAS; Simple; p.Gly12Val (c.35G>T); ClinVar=VCV000012583; Zygosity=Heterozygous (PubMed=11169959; PubMed=11787853; PubMed=15367885; Cosmic-CLP).<br>• Mutation; HGNC; 11998; TP53 Simple; p.Cys242Arg (c.724T>C); ClinVar=VCV000449512; Zygosity=Homozygous (PubMed=11169959; PubMed=11787853; PubMed=15367885; Cosmic-CLP).

D

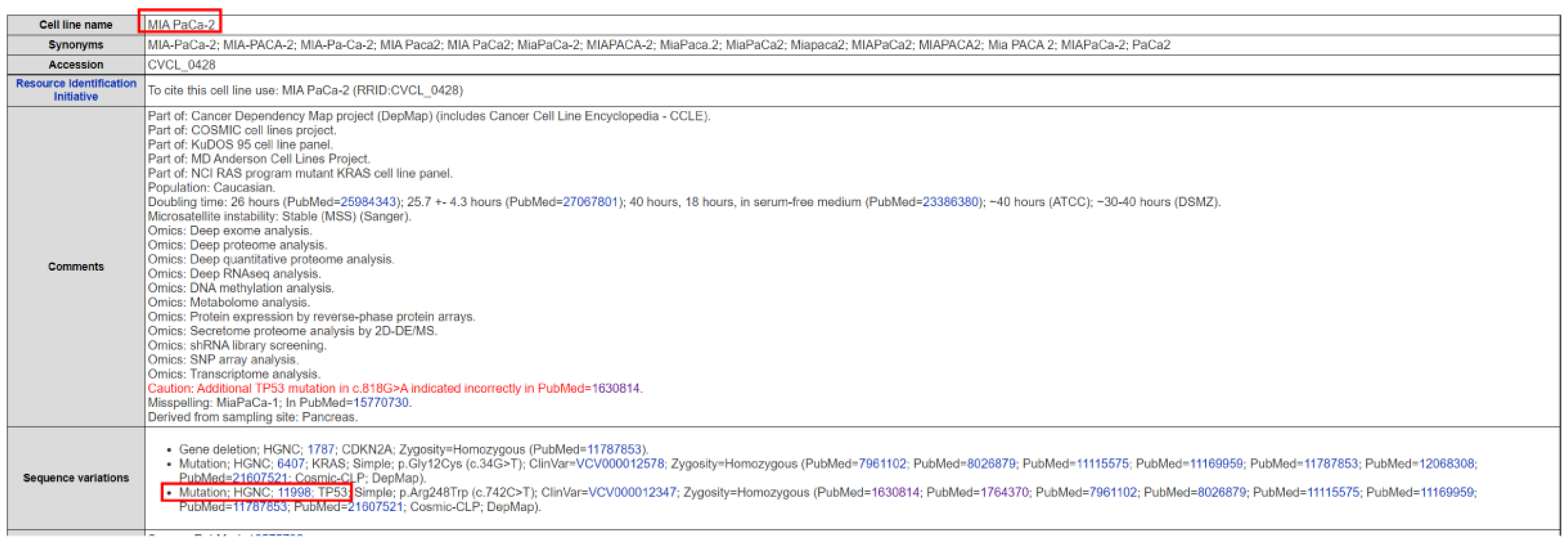

Cell line name	MIA PaCa-2
Synonyms	MIA-PaCa-2; MIA-PACA-2; MIA-Pa-Ca-2; MIA Paca2; MIA PaCa2; MiaPaCa-2; MIAPACA-2; MiaPaca.2; MiaPaCa2; Miapaca2; MIAPaCa2; MIAPACA2; Mia PACA 2; MIAPaCa-2; PaCa2
Accession	CVCL_0428
Resource Identification Initiative	To cite this cell line use: MIA PaCa-2 (RRID:CVCL_0428)
Comments	Part of: Cancer Dependency Map project (DepMap) (includes Cancer Cell Line Encyclopedia - CCLE).<br>Part of: COSMIC cell lines project.<br>Part of: KuDOS 95 cell line panel.<br>Part of: MD Anderson Cell Lines Project.<br>Part of: NCI RAS program mutant KRAS cell line panel.<br>Population: Caucasian.<br>Doubling time: 26 hours (PubMed=25984343); 25.7 +- 4.3 hours (PubMed=27067801); 40 hours, 18 hours, in serum-free medium (PubMed=23386380); ~40 hours (ATCC); ~30-40 hours (DSMZ).<br>Microsatellite instability: Stable (MSS) (Sanger).<br>Omics: Deep exome analysis.<br>Omics: Deep proteome analysis.<br>Omics: Deep quantitative proteome analysis.<br>Omics: Deep RNAseq analysis.<br>Omics: DNA methylation analysis.<br>Omics: Metabolome analysis.<br>Omics: Protein expression by reverse-phase protein arrays.<br>Omics: Secretome proteome analysis by 2D-DE/MS.<br>Omics: shRNA library screening.<br>Omics: SNP array analysis.<br>Omics: Transcriptome analysis.<br>Caution: Additional TP53 mutation in c.818G>A indicated incorrectly in PubMed=1630814.<br>Misspelling: MiaPaCa-1; In PubMed=15770730.<br>Derived from sampling site: Pancreas.
Sequence variations	• Gene deletion; HGNC; 1787; CDKN2A; Zygosity=Homozygous (PubMed=11787853).<br>• Mutation; HGNC; 6407; KRAS; Simple; p.Gly12Cys (c.34G>T); ClinVar=VCV000012578; Zygosity=Homozygous (PubMed=7961102; PubMed=8026879; PubMed=11115575; PubMed=11169959; PubMed=11787853; PubMed=12068308; PubMed=21607521; Cosmic-CLP; DepMap).<br>• Mutation; HGNC; 11998; TP53; Simple; p.Arg248Trp (c.742C>T); ClinVar=VCV000012347; Zygosity=Homozygous (PubMed=1630814; PubMed=1764370; PubMed=7961102; PubMed=8026879; PubMed=11115575; PubMed=11169959; PubMed=11787853; PubMed=21607521; Cosmic-CLP; DepMap).

E

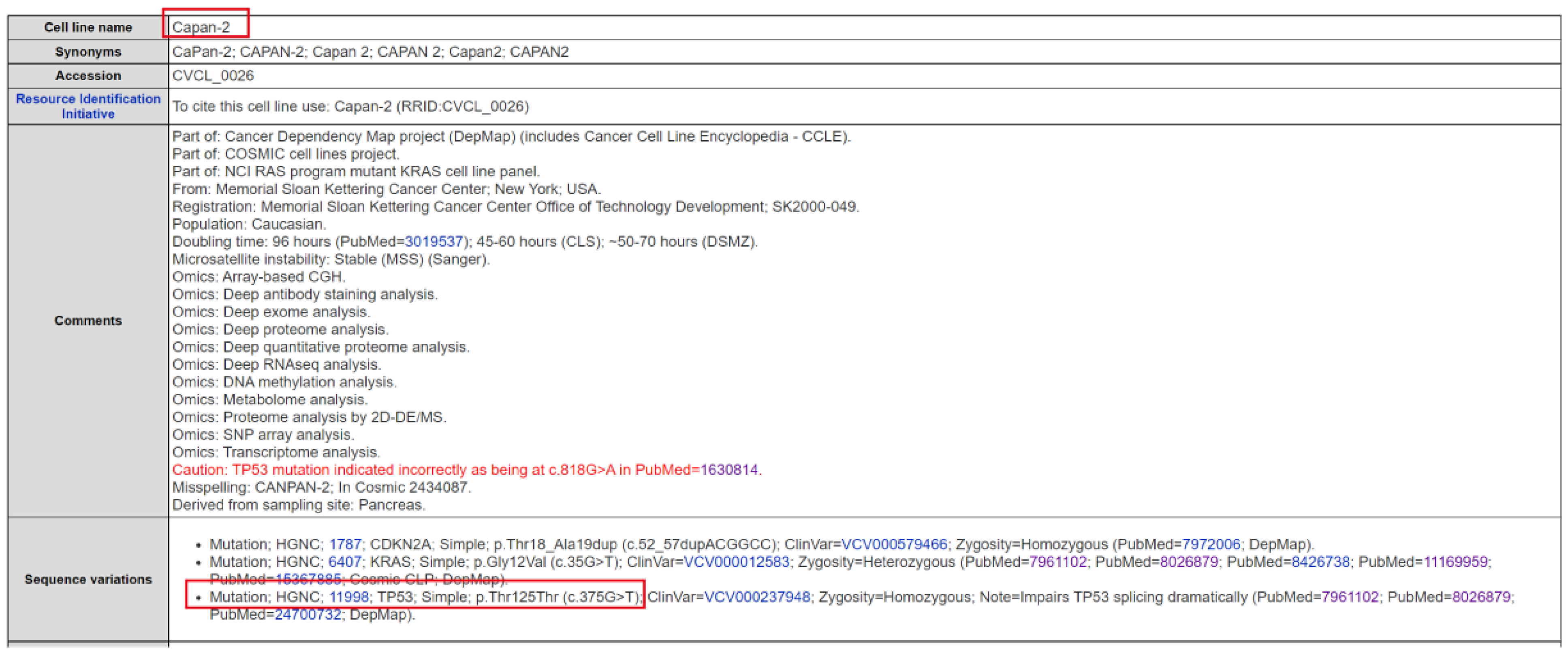

Cell line name	Capan-2
Synonyms	CaPan-2; CAPAN-2; Capan 2; CAPAN 2; Capan2; CAPAN2
Accession	CVCL_0026
Resource Identification Initiative	To cite this cell line use: Capan-2 (RRID:CVCL_0026)
Comments	Part of: Cancer Dependency Map project (DepMap) (includes Cancer Cell Line Encyclopedia - CCLE).<br>Part of: COSMIC cell lines project.<br>Part of: NCI RAS program mutant KRAS cell line panel.<br>From: Memorial Sloan Kettering Cancer Center; New York; USA.<br>Registration: Memorial Sloan Kettering Cancer Center Office of Technology Development; SK2000-049.<br>Population: Caucasian.<br>Doubling time: 96 hours (PubMed=3019537); 45-60 hours (CLS); ~50-70 hours (DSMZ).<br>Microsatellite instability: Stable (MSS) (Sanger).<br>Omics: Array-based CGH.<br>Omics: Deep antibody staining analysis.<br>Omics: Deep exome analysis.<br>Omics: Deep proteome analysis.<br>Omics: Deep quantitative proteome analysis.<br>Omics: Deep RNAseq analysis.<br>Omics: DNA methylation analysis.<br>Omics: Metabolome analysis.<br>Omics: Proteome analysis by 2D-DE/MS.<br>Omics: SNP array analysis.<br>Omics: Transcriptome analysis.<br>Caution: TP53 mutation indicated incorrectly as being at c.818G>A in PubMed=1630814.<br>Misspelling: CANPAN-2; In Cosmic 2434087.<br>Derived from sampling site: Pancreas.
Sequence variations	• Mutation; HGNC; 1787; CDKN2A; Simple; p.Thr18_Ala19dup (c.52_57dupACGGCC); ClinVar=VCV000579466; Zygosity=Homozygous (PubMed=7972006; DepMap).<br>• Mutation; HGNC; 6407; KRAS; Simple; p.Gly12Val (c.35G>T); ClinVar=VCV000012583; Zygosity=Heterozygous (PubMed=7961102; PubMed=8026879; PubMed=8426738; PubMed=11169959; PubMed=15367885; Cosmic-CLP; DepMap).<br>• Mutation; HGNC; 11998; TP53; Simple; p.Thr125Thr (c.375G>T); ClinVar=VCV000237948; Zygosity=Homozygous; Note=Impairs TP53 splicing dramatically (PubMed=7961102; PubMed=8026879; PubMed=24700732; DepMap).

Figure. S1 Knocking down LINC00460 promotes cell apoptosis. A The cell apoptosis rate of PANC1 while knocking down LINC00460. **B** The cell apoptosis rate of CFPAC while knocking down LINC00460. **C** The cell apoptosis rate of MiaPaCa2 while overexpressing LINC00460. **D** The protein level of BAX, Caspase3 and Caspase8 while knocking down LINC00460 in PANC1 and CFPAC or overexpressing LINC00460 in MiaPaCa2. ***P<0.001

Figure S2 A The cell numbers of G1, S and G2 phase while knocking down LINC00460 or not in PANC1. **B** The cell numbers of G1, S and G2 phase while knocking down LINC00460 or not in CFPAC. **C** The protein level of CDK4 and CDK6 while knocking down LINC00460 in PANC1 and CFPA or overexpressing LINC00460 in MiaPaCa2. **D** The protein level of Ki-67, N-cad and E-cad while knocking down LINC00460 in PANC1 and CFPAC or overexpressing LINC00460 in MiaPaCa2. *P<0.05; **P<0.01; ***P<0.001

Figure S3 A The sequence logo of HIF-1α in JASPAR database. **B** The correlation analysis of USP10 and HIF-1α using data from TCGA. **C** The correlation analysis of UBE2V1 and HIF-1α using data from TCGA. **D** The protein level of HIF-1α while knocking down HIF-1α in PANC1, CFPAC and MiaPaCa2 cells. **E** The mRNA expression of HIF-1α while knocking down HIF-1α in PANC1, CFPAC and MiaPaCa2 cells. **F** The mRNA expression of LINC00460 while knocking down HIF-1α in PANC1 and CFPAC cells. *P<0.05; **P<0.01; ***P<0.001

Figure S4 Knocking down LINC00460 could reverse the malignant biological behavior induced by hypoxia. PANC1 and CFPAC cells were treated with normoxia, hypoxia, a combination of si-HIF-1α and hypoxia, a combination of si-LINC00460 and hypoxia, a combination of si-HIF-1α co-transfected overexpression LINC00460 and hypoxia. The results of CCK8 assays in PANC1 (**A**) and CFPAC (**B**). The representative images and results of transwell assays in PANC1 (**C**) and CFPAC (**D**). The representative images of wound healing assays and the results of wound closure rate after 24h in PANC1 (**E**)and CFPAC (**F**). *P<0.05; **P<0.01; ***P<0.001

Figure S5 A The location of U6, 18S and LINC00460 in PANC1. **B** The location of U6, 18S and LINC00460 in CFPAC. **C** Relative expression of miR-4689, miR-4649-3p and miR-6858-5p in PANC1 knocked down LINC00460. **D** Relative expression of miR-4689, miR-4649-3p and miR-6858-5p in CFPAC knocked down LINC00460 **E** Relative expression of miR-4689, miR-4649-3p and miR-6858-5p in MiaPaCa2 overexpressed LINC00460. F Relative expression of SH3TC, UBE2V1, TRIM71, TNS1, CACNA1B, UNC119B and CDKN1A after transfection of control vector, miR-4689 mimics and miR-4689 inhibitor. **G** Correlation analysis of LINC00460 and UBE2V1 using data from TCGA. **H** Correlation analysis of LINC00460 and UBE2V1 using data from our center. *P<0.05; **P<0.01; ***P<0.001

Figure S6 A The mRNA expression of UBE2V1 when transfected si-NC and si-UBE2V1 in Capan2 cells. **B** The result of CCK8 assay in Capan2 cells. **C** The representative images and results of colony formation assay in Capan2 cells. **D** The representative images and results of transwell assays in Capan2 cells. **E** The representative images of wound healing assays and the results of wound closure rate after 24h in Capan2 cells. *P<0.05; **P<0.01; ***P<0.001

Figure S7 A The transcripts level of USP10 in 178 tumor tissues and 171 normal tissues of PAAD patients. B The expression of USP10 in 179 tumor tissues and 171 normal tissues of PAAD patients. C Correlation analysis of USP10 and p53 using data from TCGA. D Overall survival analysis of USP10 in pancreatic cancer from TCGA database. E Disease free survival analysis of USP10 in pancreatic cancer from TCGA database.

Figure S8 A The mRNA expression of USP10 while knocking down USP10 or not in PANC1 cells. **B** The mRNA expression of USP10 while knocking down USP10 or not in CFPAC cells. **C** The correlation analysis of USP10 and p53 using data from TCGA. **D** CoIP assays using USP10 and p53 antibodies to verify the combination of USP10 and p53 in PANC1. **E** CoIP assays using USP10 and p53 antibodies to verify the combination of USP10 and p53 in Capan2. *P<0.05; **P<0.01; ***P<0.001

Figure S9 A The mRNA expression of USP10 when transfected si-NC and si-UBE2V1 in Capan2 cells. **B** The mRNA expression of p53 when transfected si-NC and si-UBE2V1 in Capan2 cells. **C** The result of CCK8 assay in Capan2 cells. **D** The representative images and results of transwell assays in Capan2 cells. **E** The representative images of wound healing assays and the results of wound closure rate after 24h in Capan2 cells. *P<0.05; **P<0.01; ***P<0.001

Figure S10 A Mutation information of p53 in PANC1, CFPAC and Capan2 in ATCC website. **B** Mutation information of p53 in PANC1 in Cellosaurus website. **C** Mutation information of p53 in CFPAC in Cellosaurus website. **D** Mutation information of p53 in MiaPaCa2 in Cellosaurus website. **E** Mutation information of p53 in Capan2 in Cellosaurus website.
